# CFAP251 Deficiency Induces Male Infertility and PCD-like Ciliary Defects by Disrupting TUBB4B and SLC25A4 Recruitment in Humans and Mice

**DOI:** 10.7150/ijbs.128822

**Published:** 2026-02-26

**Authors:** Liting Liu, Zhanyu Wang, Yiling Zhou, Dongdong Tang, Rong Hua, Kuokuo Li, Guanxiong Wang, Fan Yang, Chunyu Liu, Yunxia Cao, Huan Wu, Yang Gao, Xiaojin He

**Affiliations:** 1 Reproductive Medicine Center, Department of Obstetrics and Gynecology, the First Affiliated Hospital of Anhui Medical University, Hefei 230022, China.; 2 Reproductive Medicine Center, Department of Obstetrics and Gynecology, Shanghai General Hospital, Shanghai Jiao Tong University School of Medicine, No. 650 Xinsongjiang Road, Shanghai 200080, China.; 3 NHC Key Laboratory of Study on Abnormal Gametes and Reproductive Tract (Anhui Medical University), Hefei 230032, China.; 4 Department of Obstetrics and Gynecology, Fuyang Hospital of Anhui Medical University, Fuyang 236112, China.; 5 International Peace Maternity and Child Health Hospital, Shanghai Jiao Tong University School of Medicine, Shanghai 200030, China.; 6 Shanghai Key Laboratory of Embryo Original Diseases, Shanghai 200030, China.

**Keywords:** CFAP251, male infertility, MMAF, PCD, ICSI

## Abstract

Mutations in the *Cfap251* gene have been identified as causative for morphology and motility abnormalities in spermatozoa of infertile males, manifesting as multiple morphological abnormalities of the sperm flagella (MMAF). However, the mechanism underlying CFAP251-associated MMAF remains poorly understood, and the role of CFAP251 deficiency in PCD remains unclear. This study aimed to elucidate the pathogenic mechanism linking CFAP251 deficiency to human male infertility and to determine whether CFAP251 plays an active role in both flagella and cilia across species. A *Cfap251* knockout mouse line was generated on a C57Bl/6J strain. *CFAP251* mutations were identified by whole-exome sequencing on probands from Han Chinese families with primary infertility and MMAF. The *Cfap251* knockout mouse model replicated the MMAF and male infertility phenotypes observed in humans for the first time. Moreover, the *Cfap251* knockout mouse and one patient showed PCD-like symptoms, including tinnitus, seasonal cough, and radiological findings of coarsened lung markings and solitary pulmonary bulla associated with chronic bronchitic inflammation. Mechanistically, CFAP251 was confirmed to interact with TUBB4B, and its disruption led to the downregulation of TUBB4B, impairing spermiogenesis and ciliary function. Furthermore, CFAP251 was found to interact with the mitochondrial protein SLC25A4, suggesting a role in regulating energy transport. Favorable outcomes following ICSI were observed, with the successful birth of healthy offspring in four patients caused by *CFAP251* variants. These included two cases with two novel homozygous splice-altering variants (c.1535+1G>C and c.1269+2T>C) and two previously reported MMAF cases. Our findings highlight the underlying risk of male infertility and PCD-like ciliary defects associated with *CFAP251* and provide a valuable reference for personalized genetic counselling and clinical treatment of affected individuals.

## Introduction

Infertility has become a global health concern affecting approximately 15% of couples, with male infertility accounting for approximately half of all cases 1. Asthenoteratozoospermia is a prevalent condition underlying male infertility. Multiple morphological abnormalities of the flagella (MMAF) represent a distinct subtype of asthenoteratozoospermia characterized by severe genetic defects 2. Recent investigations have revealed several gene mutations associated with MMAF, including those in the dynein axonemal heavy chain (*DNAH*), coiled-coil domain-containing, and cilia and flagellum-associated protein (CFAP) families.

Motile cilia and flagella are evolutionarily conserved organelles with axonemal structures consisting of two central pairs (CPs) and nine peripheral doublet microtubules (DMTs), connected by radial spokes (RSs) 3. The *CFAP* gene family encodes proteins that regulate ciliary and flagellar motility via coordinated control of axoneme assembly, signaling networks, and transport systems, with dysfunction directly linked to infertility and ciliopathies. Mutations in several *CFAP* genes have been linked to MMAF, with or without primary ciliary dyskinesia (PCD) symptoms. Currently, 19 members of the *CFAP* gene family have been reported, among which 14 genes (*CFAP43-45, CFAP47, CFAP52, CFAP54, CFAP57-58, CFAP61, CFAP65, CFAP69-70, CFAP74, CFAP91,* and *CFAP251*) are associated with male infertility 4, and 5 (*CFAP53, CFAP74, CFAP221, CFAP298*, and *CFAP300*) are associated with PCD 5, 6. Clinical and molecular evidence have confirmed that pathogenic variants in *CFAP45, CFAP52,* and *CFAP54* concurrently induce PCD and impaired spermatogenesis 7, 8. The substantial genetic heterogeneity and broad clinical spectrum of *CFAP* genes highlight their functional uniqueness and mechanistic complexity in ciliary and flagellar biology. However, the susceptibility of uncharacterized cilia- and flagellum-associated factors related to MMAF or PCD remains unclear owing to the absence of human genetic evidence or corresponding animal models.

Among the CFAP family, CFAP251 is one of the less characterized members. CFAP251, also known as WDR66, is a component of the calmodulin- and radial-spoke-associated complex located adjacent to DNAH1 in *Tetrahymena thermophila* at the base of radial spoke 3 (RS3) in the RS complex 9. A direct physical link between CFAP251, IDA3, and RS3 has been demonstrated in co-immunoprecipitation (Co-IP) experiments 10. Mutations in *CFAP251* have been implicated in male infertility; however, pathogenic evidence remains limited, and the underlying mechanism remains unclear 11. Furthermore, although CFAP251 expression is predominantly detected in the trachea and testes of mammals, its physiological functions remain largely elusive. Therefore, we investigate its wider biological role in ciliary and flagellar motility.

In this study, we generated *Cfap251* knockout mice, which replicated the MMAF and male infertility phenotypes observed in humans, and demonstrated that Cfap251 deficiency led to severe axonemal defects in sperm flagella, while subtle defects were observed in cilia. In mouse testes, CFAP251 interacts with microtubule protein TUBB4B and mitochondrial-related protein SLC25A4, and Protein-protein Complexes Structure Modeling and Binding Affinity Prediction assays further corroborated the potential molecular interaction interfaces between CFAP251 and these two proteins. Molecular assays demonstrated that CFAP251 is a conserved protein across species, with distribution patterns recapitulating those of intramanchette transport and intraflagellar transport-dependent assembly mechanisms in spermatids. Our findings revealed that the absence of CFAP251 causes general axonemal disruption, which indirectly leads to the mislocalization of axonemal components in both the flagella and cilia. Furthermore, we demonstrated that biallelic mutations in *CFAP251* cause male infertility with a PCD-like phenotype in both humans and mice. These findings enhance our understanding of the development of axonemal structures in flagella and cilia. They provide valuable insights for genetic counseling and individualized treatment of male infertility associated with *CFAP251* mutations.

## Materials and Methods

### Study participants

Two Han Chinese men with MMAF (F1 IV-1 and F2 II-1) were recruited from the asthenoteratozoospermia cohort at the First Affiliated Hospital of Anhui Medical University. Both patients had a history of primary infertility for more than 1 year. Additionally, the pregnancy outcomes of two previously reported MMAF cases (M1 IV-1 and M4 II-1) were investigated. Two men with normal semen analysis results were enrolled as controls. This study was approved by the Ethics Committee of the First Affiliated Hospital of Anhui Medical University (Hefei, China). All participants provided written informed consent.

### Whole-exome sequencing (WES), bioinformatic analysis, and Sanger sequencing

Blood samples were collected from patients for DNA extraction. WES and bioinformatic analyses were performed as previously described 12. Briefly, sequencing was conducted using the Illumina HiSeq platform, and the raw FASTQ data were mapped to the human genome using BWA software. The allele frequency databases (1000G, EXAC03_EAS, and gnomAD_exome_EAS) and deleterious prediction tools (SIFT, PolyPhen-2, mutations Taster, and CADD) were used to annotate the variants. Variants with an allele frequency of <0.01 were retained, and analysis focused on loss-of-function (including splicing [≤2 bp], stop-gain, stop-loss, and frameshift indels) and deleterious missense variants (predicted by at least three of the four abovementioned software). Sanger sequencing was used to validate the inheritance patterns of variants. The primers used are listed in Supplementary Table S1.

### *Cfap251* knockout mouse model generation with CRISPR-Cas9

A knockout mouse model harboring the frameshift variant of *Cfap251* (NCBI: NM_144668) was generated using the CRISPR-Cas9 technology. The sequence of the protospacer-adjacent motif was CCT. A single-guide RNA was designed for *Cfap251* exon9 and its sequence was GGTTGGACTTCTCGGTTGGC. A frameshift variant of *Cfap251* was identified using Sanger sequencing in the founder mouse and its offspring (the primer information is provided in Supplementary Table S2). All mouse experiments were performed in accordance with the US National Institutes of Health's Guide for the Care and Use of Laboratory Animals, and the study was approved by the Animal Ethics Committee of Anhui Medical University. Adult mice (≥8 weeks old) were used for subsequent experiments.

### Routine semen analysis and Papanicolaou staining of spermatozoa

Routine semen analysis was performed using the SCA Sperm Quality Detection System (version 5.1; Microptic, Spain) 13. Sperm morphology was assessed by modified Papanicolaou staining, with at least 200 spermatozoa examined during the analysis 14. Routine semen analysis and Papanicolaou staining of spermatozoa were performed as previously described and according to the WHO standards for human semen examination and processing (6th ed.).

### Scanning electron microscopy (SEM) and transmission electron microscopy (TEM)

For SEM and TEM, spermatozoa were prepared as previously described 15. For SEM, samples were examined using field-emission SEM (Nova Nano 450; Thermo Fisher Scientific). For TEM, the samples were observed using cryoelectron microscopy (Tecnai G2 Spirit 120 kV).

### Immunofluorescence analysis

Spermatozoa were fixed in 4% paraformaldehyde, mounted on slides, and incubated with primary antibodies overnight at 4 °C. Subsequently, slides were incubated with secondary antibodies for 1-2 h at 37 °C. Finally, slides were observed and photographed using an LSM980 confocal microscope (Carl Zeiss AG). All antibodies used are listed in Supplementary Table S3.

### Western blotting

Proteins were extracted from samples, heated at 100 °C for 10 min, separated with 10% SDS-polyacrylamide gel electrophoresis (SDS-PAGE), and transferred onto polyvinylidene fluoride membranes. The membranes were blocked with 5% diluted milk in TBST (TBS containing 0.1% Tween-20) and sealed for 2 h at 37 °C, then incubated overnight at 4 °C with primary antibodies diluted in TBST. After washing with TBST, membranes were incubated with secondary antibodies for 1-2 h at room temperature. Enhanced chemiluminescence (BL520A, Biosharp) was used for visualization. The reference protein β-actin was used as a loading control. All antibodies used are listed in Supplementary Table S3.

### Immunofluorescence of the testicular germ cells

Mouse testes were dissected and immediately fixed in 4% paraformaldehyde containing 0.05% Triton X-100 in PBS for 5 min at room temperature. The fixed tissue was transferred onto a slide, overlaid with a coverslip, and gently compressed before freezing in liquid nitrogen for 10 min. Slides were then stored at -80 °C prior to immunofluorescence analysis. For staining, coverslips were removed, and slides were washed three times in PBS, permeabilized with 0.1% Triton X-100 in PBS for 10 min, and washed again three times in PBS. Non-specific binding sites were blocked using 5% bovine serum albumin (Sangon Biotech, A500023-0005). Tissue sections were incubated with primary antibodies overnight at 4 °C, followed by incubation with the appropriate secondary antibodies. Nuclei were visualized using DAPI counterstaining. Immunofluorescence images were acquired using a Zeiss LSM 980 confocal microscope.

### Quantitative real-time PCR (qRT-PCR)

To investigate the pathogenic effects of the *CFAP251* variant, real-time PCR (RT-PCR) was performed to measure *CFAP251* mRNA expression. Total RNA was isolated using TRIzol reagent (Invitrogen) and reverse-transcribed into complementary DNA (cDNA) using the PrimeScript RT Reagent Kit (Takara). We then performed qRT-PCR using qPCR SYBR Green Master Mix (Vazyme), with β-actin as an internal control. Primers used are listed in Supplementary Table S4.

### Tissue collection and histological analysis

The testes and cauda epididymis of *Cfap251^+/+^* and *Cfap251^-/-^* mice were dissected immediately after euthanasia. Samples were fixed in 4% paraformaldehyde (Solarbio, P1110) for at least 24 h, dehydrated through a graded ethanol series, made transparent in xylene, immersed in melted paraffin, and embedded in paraffin. For histological analysis, 5-μm sections were mounted on slides and stained with periodic acid-Schiff (PAS; Solarbio) and hematoxylin and eosin (H&E; Solarbio) to determine spermatogenic epithelial cycle stages and spermatid development in testes and cauda epididymis.

### Immunoprecipitation-mass spectrometry

Immunoprecipitation was performed using protein extracts from *Cfap251^+/+^* mouse testes (P60) lysed in NP-40 lysis buffer (Biosharp, BL653A) supplemented with 1% protease inhibitor (100 mM; Thermo Fisher Scientific). CFAP251 and rabbit IgG antibodies were incubated with protein A/G magnetic beads (Thermo Fisher Scientific). Immunoprecipitated proteins from the IgG and CFAP251 groups were separated by SDS-PAGE and stained using a Fast Silver Stain Kit (Beyotime). The separated peptides were analyzed on a Q Exactive mass spectrometer (Thermo Fisher) for 60 min. MS data files were processed for protein identification, specifically for the mice, using MaxQuant 2.0.1.0.

### Plasmid construction

Full-length human *CFAP251* cDNA (NM_144668.6) was synthesized and cloned into the 3×Flag pcDNA3.1 vector to generate the Flag-tagged CFAP251 plasmid. The plasmid encoding human *SLC25A4* (NM_001151.4) was synthesized and cloned into the 3×Myc pcDNA3.1 vector to produce the Myc-tagged SLC25A4 plasmid. Human *TUBB4B* cDNA (NM_006088.6), *CABYR* cDNA (NM_153769.3) and *AKAP3* cDNA (NM_001278309.2) were synthesized and cloned into the pEGFP-C1 vector to generate GFP-tagged TUBB4B plasmids. All constructed plasmids were verified by DNA sequencing.

### Cell culture and transfections

HEK293T cells were cultured in Dulbecco's modified Eagle medium containing penicillin-streptomycin and 10% fetal bovine serum. Lipofectamine 3000 (Invitrogen) was used to transfect cells with the appropriate plasmids.

### Co-IP experiments

For the Co-IP assays, extracted total proteins were incubated overnight at 4 °C with the appropriate primary antibody, followed by incubation with protein A/G magnetic beads (Thermo Fisher Scientific, 88804) for 3 h at 4 °C. After washing with NP-40 and eluting with 2× SDS loading buffer, the samples were boiled for 10 min at 95 °C and analyzed by immunoblotting assay.

### Intracytoplasmic sperm injection (ICSI) in mice

ICSI was administered as previously described 16, with similar culture conditions for the oocyte after injection. To calculate fertilization, cleavage, and embryo formation rates, we recorded the number of male and female pronuclei formed after 6 h, 2-cell embryos formed after 24 h, and typical blastocysts formed after 96 h following sperm injection. The rates were calculated using the following formulas: Fertilization Rate = (number of male and female pronuclei / total number of injected oocytes) × 100%, Cleavage Rate = (number of 2-cell embryos / number of male and female pronuclei) × 100%, and Embryo Formation Rate = (number of blastocysts / number of male and female pronuclei) × 100%.

### ICSI in patients

The spouses of F1 IV-1, F2 II-1, M1 IV-1, and M4 II-1 underwent standard controlled ovarian hyperstimulation and oocyte retrieval, followed by ICSI. The embryos were cultured to day 5 or day 6, and viable thawed embryos were transferred to the respective partners. Clinical pregnancy was confirmed by ultrasonography 28 days after embryo transfer.

### Protein-protein Complexes Structure Modeling and Binding Affinity Prediction

Human-specific protein sequences for CFAP251 (UniProt ID: Q8TBY9), TUBB4B (UniProt ID: P68371), and SLC25A4 (UniProt ID: P12235) were retrieved from the UniProt database. Full-length sequences of these proteins were then submitted to AlphaFold3 for structure prediction, generating high-confidence models for each protein based on their respective sequences. The predicted protein structures were analyzed using PRODIGY to calculate the binding affinity (ΔG) of protein-protein interactions. A more negative ΔG value indicates a more stable binding. PyMOL was used to visualize the 3D structures of the protein complexes and analyze the binding interfaces. Detailed examination of the interaction surfaces identified key residues involved in hydrophobic interactions, hydrogen bonds, and electrostatic interactions.

### Quantification and statistical analysis

Statistical analyses and parameters, including significance levels, are presented in the figure legends. All analyses were performed using GraphPad Prism software. Comparisons were conducted using Student's t-test, and a P-value <0.05 was considered statistically significant.

## Results

### CFAP251 protein is conserved in mammals and highly expressed in the testis and bronchus

**Figure 1:**
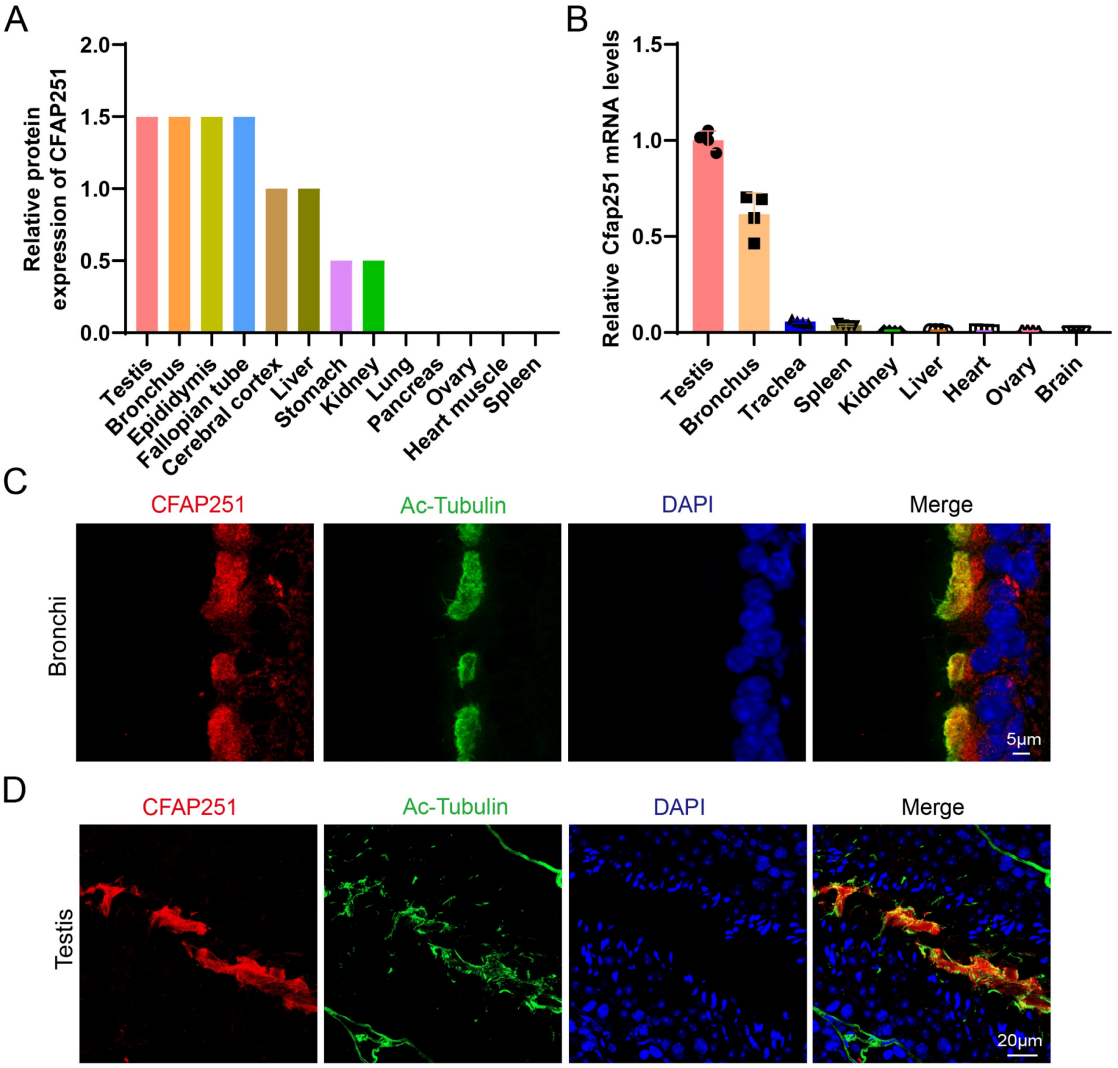
** Tissue distribution and subcellular localization of CFAP251. (A)** The relative protein abundance of CFAP251 across human tissues from the Human Protein Atlas database (https://www.proteinatlas.org/), showing predominant expression in the testis, bronchus, epididymis, and fallopian tube, with low or negligible levels in other organs. **(B)** Relative *Cfap251* mRNA expression measured by qRT-PCR, normalized to housekeeping genes, demonstrating enriched transcript levels in the testis and bronchus, and minimal expression in non-ciliated tissues. Gapdh was used for norminalization. Data were presented as mean±SEM, n=4 (four biological replicates) **(C)** Immunofluorescence staining of bronchial epithelial cells showing CFAP251 (red) colocalized with acetylated tubulin (green), a marker of motile cilia; nuclei were counterstained with DAPI (blue). Scale bar, 5 µm. **(D)** Immunofluorescence analysis of seminiferous tubules in the testis, revealing CFAP251 localization along the sperm flagellum co-stained with acetylated-tubulin (green); nuclei were counterstained with DAPI (blue). Scale bar, 20 µm.

Analysis using the Human Protein Atlas (https://www.proteinatlas.org/ENSG00000158023-CFAP251) revealed that CFAP251 is broadly expressed in ciliated and flagellated cells, with particularly high enrichment in the human testis and bronchus (Figure 1A). Moreover, we detected *Cfap251* expression in multiple mouse organs via RT-PCR (Figure 1B). This pattern is consistent with that observed in humans, suggesting a conserved function. To validate these findings, we performed immunofluorescence staining on mouse paraffin sections derived from these tissues, both of which contain “9+2” motile axonemes. In the respiratory epithelium, CFAP251 was detected in the ciliary axoneme and cytoplasm (Figure 1C), whereas in the testis, CFAP251 was localized to the sperm flagellum within the seminiferous tubules (Figure 1D). These results demonstrate that CFAP251 expression is conserved between humans and mice. Consistent with its expression pattern, CFAP251 is likely essential for sperm motility and may also play a role in regulating ciliary motility through its involvement in axonemal assembly.

### Deletion of *Cfap251* causes sperm abnormalities and male infertility in mice

**Figure 2:**
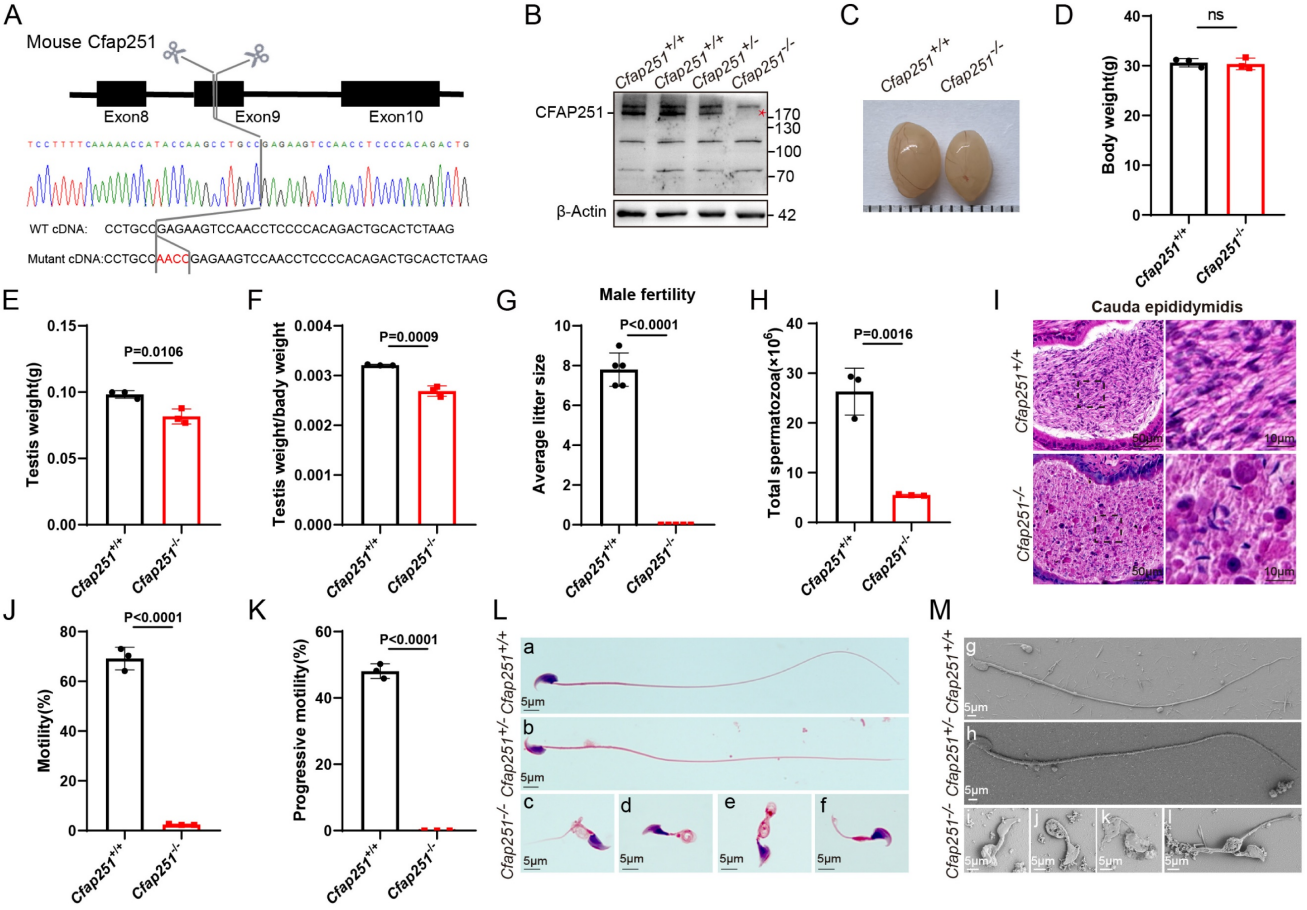
** Generation and phenotypic characterization of *Cfap251* knockout mice. (A)** Schematic representation of the CRISPR-Cas9-mediated deletion in *Cfap251* spanning exon 9, confirmed by Sanger sequencing. **(B)** Immunoblot analysis showing loss of CFAP251 protein in *Cfap251*^-/-^ testes compared with wild-type controls, with β-actin as loading control. **(C)** Representative images of testes from *Cfap251*^+/+^ and *Cfap251*^-/-^ males. **(D)** Body weight is comparable between genotypes (n = 3 per genotype). Data are presented as mean ± SD, and statistical test is a two-tailed Student's t-test. ns, not significant. **(E, F)** Testis weight and relative testis-to-body weight ratio are significantly reduced in *Cfap251*^-/-^ mice (n = 3 per genotype). Data are presented as mean ± SD, and statistical test is a two-tailed Student's t-test. p = 0.016 and p = 0.0009. **(G)** Male fertility is severely impaired in *Cfap251*^-/-^ mice, as indicated by reduced litter size (n = 5 per genotype). Data are presented as mean ± SD, and statistical test is a two-tailed Student's t-test. p < 0.0001. **(H)** Total sperm count from the cauda epididymis is markedly decreased in knockouts (n = 3 per genotype). Data are presented as mean ± SD, and statistical test is a two-tailed Student's t-test. p = 0.0016. **(I)** H&E staining of cauda epididymis sections reveals reduced sperm accumulation in *Cfap251*^-/-^ mice. Scale bar, 10 µm. **(J, K)** Computer-assisted sperm analysis shows near-complete loss of total and progressive motility in *Cfap251*^-/-^ spermatozoa (n = 3 per genotype). Data are presented as mean ± SD, and statistical test is a two-tailed Student's t-test. p<0.0001. **(L)** Morphological assessment demonstrates normal spermatozoa in wild-type mice and multiple head and tail malformations in *Cfap251*^-/-^ sperm. **(M)** Scanning electron microscopy further confirms abnormal head and tail structures in knockout spermatozoa. Scale bar, 5 µm.

To functionally confirm the deletion of *Cfap251*, we generated *Cfap251* knockout mice using the CRISPR-Cas9 gene-editing technology. A Cfap251 gene knockout mouse model with a 4-bp deletion in exon 8 was successfully established (Figure 2A). In contrast to the observations in control mice, immunoblotting of testis lysates failed to detect Cfap251 proteins (Figure 2B), and immunofluorescence staining of testis sections did not show Cfap251 signals in *Cfap251^-/-^* mice (Supplementary Figure 1A). RT-PCR analysis of the mouse testes revealed that Cfap251 expression was initially detected on postnatal day 21 and persisted into adulthood (Supplementary Figure 1B). The timing of expression coincides with the period of spermiogenesis, which is characterized by nuclear condensation and flagellum formation. Immunofluorescence staining of sperm cell suspensions further demonstrated the dynamic localization of CFAP251 during sperm maturation; in elongating spermatids from wild-type mice, CFAP251 initially surrounded the head and subsequently migrated toward the developing flagellum (Supplementary Figure 1C). Furthermore, the body weights of *Cfap251^-/-^* and *Cfap251^+/+^* males were comparable (Figure 2C); however, smaller testes were observed in *Cfap251^-/-^* males (Figure 2D-F). Although *Cfap251^-/-^* males developed normally, they were infertile (Figure 2G). Moreover, the average sperm count per epididymis was substantially reduced in *Cfap251^-/-^* mice (Figure 2H). H&E staining of epididymal sections also showed a striking reduction in the sperm count of *Cfap251^-/-^* mice (Figure 2I). The cauda epididymal spermatozoa of *Cfap251^-/-^* males were 100% immotile compared with those of *Cfap251^+/+^* (Figure 2J and K). Notably, morphological analysis of sperm from the cauda epididymis of *Cfap251^-/-^* mice showed frequencies of head abnormalities and flagella (Figure 2L and M) similar to those observed in previously reported patients.

### Deletion of *Cfap251* impairs spermiogenesis and causes abnormal sperm heads

**Figure 3:**
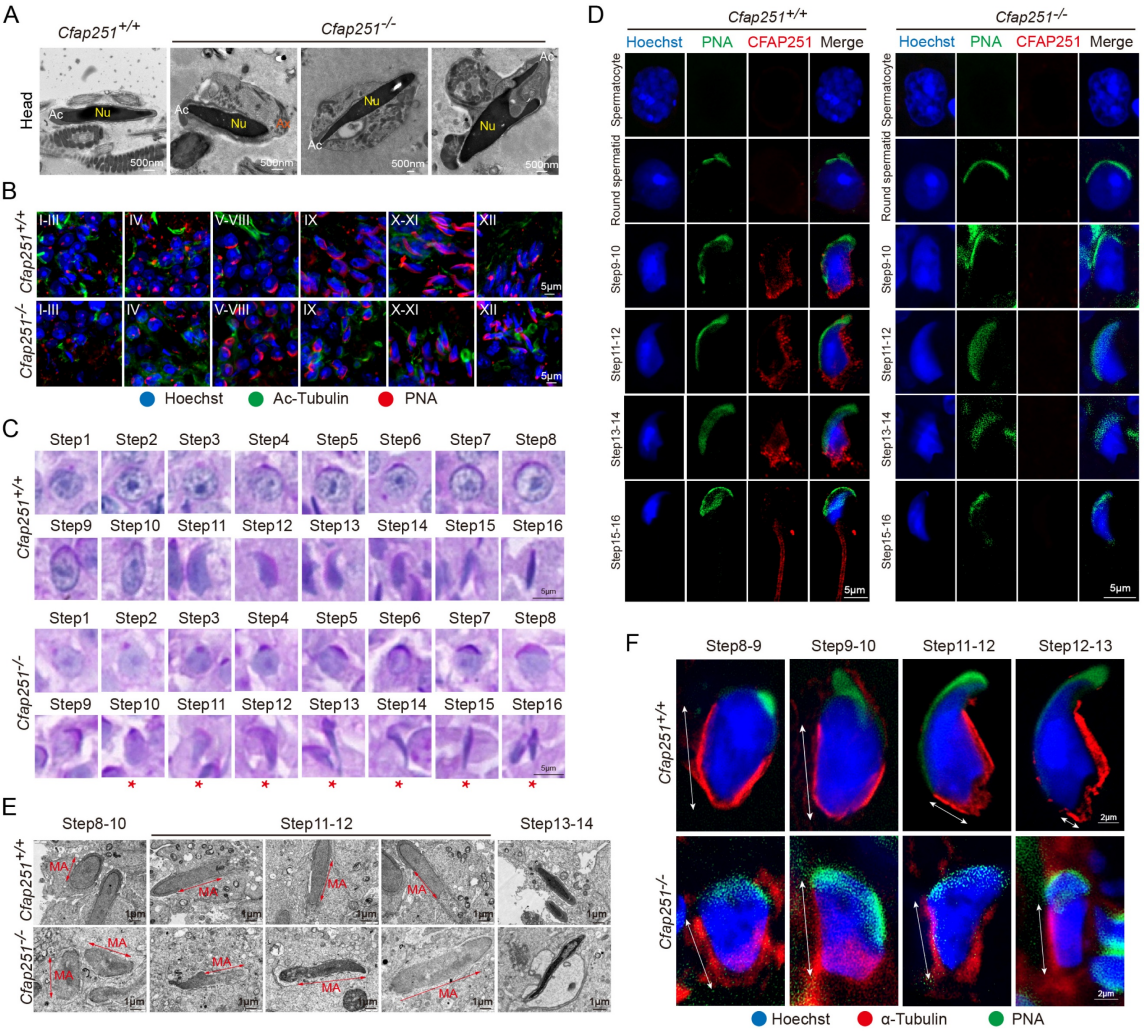
** Defective spermiogenesis, acrosome, and manchette formation in *Cfap251* knockout mice. (A)** Transmission electron microscopy (TEM) of spermatozoa from *Cfap251^+/+^* and *Cfap251^-/-^* males, showing abnormal head morphology and acrosomal defects in mutants (Ac, acrosome; Nu, nucleus; Ax, axoneme). Scale bar, 500 nm. **(B)** Immunofluorescence staining of testis sections with Hoechst (blue), acetylated tubulin (green), and PNA (red) at different seminiferous epithelial stages (I-XII), demonstrating impaired acrosome biogenesis in *Cfap251^-/-^* mice. Scale bar, 5 µm. **(C)** Periodic acid-Schiff staining of spermatids from steps 1-16, revealing aberrant acrosomal development in mutants (asterisks indicate defective steps). Scale bar, 5 µm. **(D)** Immunofluorescence of spermatids with Hoechst (blue), PNA (green), and CFAP251 (red), showing the absence of CFAP251 localization in Cfap251^-/-^ spermatids across developmental stages. Scale bar, 5µm. **(E)** TEM analysis of spermatids at steps 8-14, highlighting the disrupted manchette (MA) structure and defective manchette elongation in *Cfap251^-/-^* spermatids. Scale bar, 1 µm. **(F)** Immunofluorescence staining of developing spermatids (steps 8-13) with Hoechst (blue), α-tubulin (red), and PNA (green), showing defective manchette elongation in *Cfap251^-/-^* mice. Scale bars are indicated in each panel. Scale bar, 2 µm.

We used PAS staining to investigate spermiogenesis defects in greater detail. Mouse seminiferous tubules can be divided into 12 stages. No apparent morphological defects in Stage I-VIII tubules were observed in *Cfap251^-/-^* mice (Supplementary Figure 2A). In wild-type mice, round spermatids began to elongate at Stage IX. However, this process was largely delayed in *Cfap251^-/-^* mice, with most spermatids having insufficiently flattened nuclei and acrosomes (Supplementary Figure 2B). To understand how *Cfap251* depletion causes abnormal sperm heads, we conducted TEM on spermatozoa from the caudal epididymis of *Cfap251^+/+^* and *Cfap251^-/-^* mice. Detached or mislocated acrosomal structures were also frequently observed (Figure 3A). To characterize these defects, testicular sections were stained with peanut agglutinin (PNA), an acrosomal marker, revealing distinct acrosomal defects in *Cfap251^-/-^* mice (Figure 3B).

Additionally, PAS staining of sperm suspensions showed that the acrosomal structure was aberrantly stretched from step 10 in *Cfap251^-/-^* mice compared with that in *Cfap251^+/+^* mice (Figure 3C). We performed immunofluorescence on testicular cell suspensions to determine the cell-specific expression of CFAP251. In *Cfap251^+/+^* mice, CFAP251 expression was initially detected in step 9 round spermatids, where it was specifically localized to the post-acrosomal region, consistent with the expression pattern of the manchette structure at this stage (Figure 3D). In contrast, CFAP251 signal was absent in *Cfap251^-/-^* mice. As spermatogenesis progressed, the signal translocated to the sperm flagellum in mature spermatids at steps 15 and 16 (Figure 3D). These findings suggest that abnormal sperm head morphology is closely associated with structural defects in the manchette. TEM of the testicular sections revealed that the microtubule organization of the manchette was sparser and lacked proper structural alignment in *Cfap251^-/-^* mice than in *Cfap251^+/+^* mice (Figure 3E). These structural abnormalities were further confirmed by immunofluorescence analysis of α-tubulin and PNA staining (Figure 3F).

### CFAP251 deficiency led to abnormal mitochondrial sheath (MS) structure with mislocated annulus

**Figure 4:**
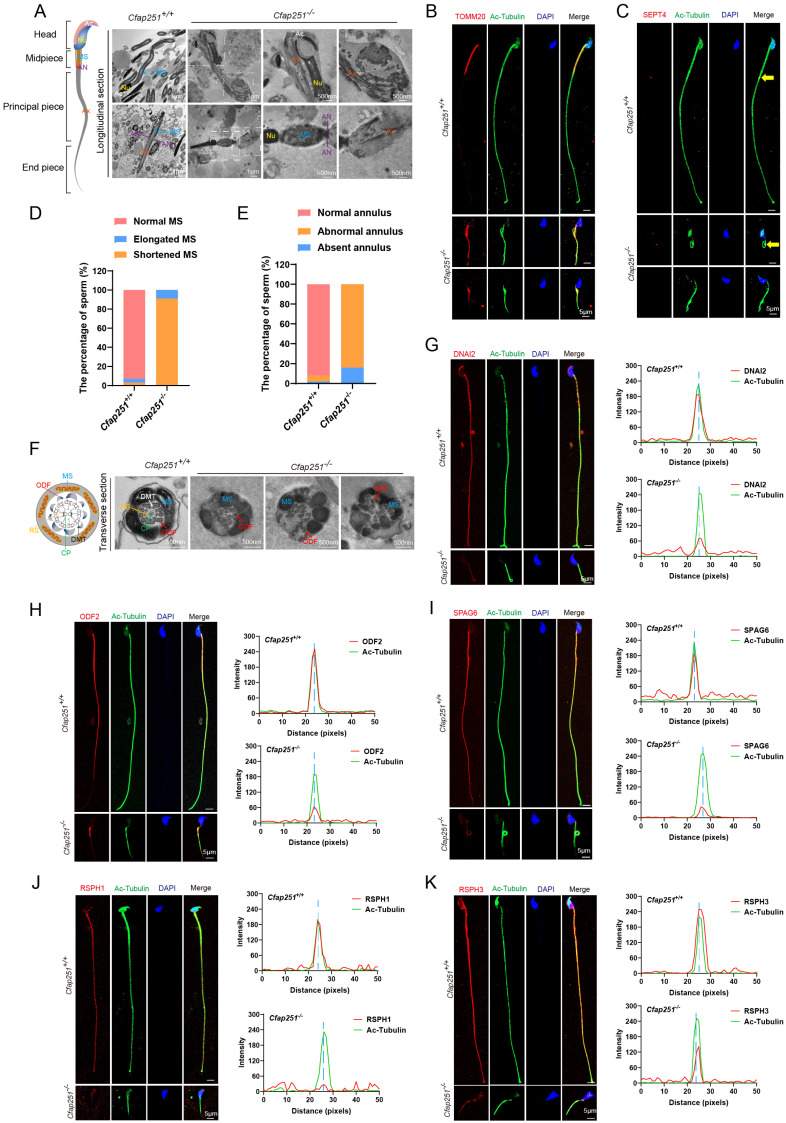
** Structural and molecular defects of the sperm flagellum in *Cfap251* knockout mice. (A)** Transmission electron microscopy (TEM) reveals structural defects in the midpiece, principal piece, and annulus of the mutant sperm. (Ac, acrosome; Nu, nucleus; MS, mitochondrial sheath; AN, annulus; Ax, axoneme). Scale bar: 200 nm, 1 µm. **(B, C)** Immunofluorescence of sperm stained with TOMM20 (B, red) or SEPT4 (C, red), acetylated tubulin (green), and DAPI (blue), indicating mitochondrial and annulus defects in *Cfap251^-/-^* sperm. Scale bar, 5 µm. **(D-E)** Enumeration analysis of fluorescence signals from A and B: the fraction of spermatozoa with elongated or shortened MS (B) and with abnormal or absent annulus phenotypes (C) was determined by counting spermatozoa (n = 100 per group), with fractions calculated as spermatozoa of each type divided by the total count, revealing a higher proportion of these phenotypes in *Cfap251^-/-^* sperm. **(F)** TEM analysis of transverse sections of the sperm flagella, revealing disrupted axoneme and periaxonemal structures. (DMT, outer doublet microtubules; MS, mitochondrial sheath; CP, central pair; RS, radial spokes; ODF, outer dense fibers). Scale bar, 500 nm. **(G-K)** Immunofluorescence of sperms with antibodies against DNAI2 (G), ODF2 (H), SPAG6 (I), RSPH1 (J), and RSPH3 (K) (red), together with acetylated tubulin (green) and DAPI (blue). Line-scan analyses (right) show reduced intensity of these axonemal proteins in *Cfap251^-/-^* sperm compared with controls. Scale bars are indicated in each panel. Scale bar, 5 µm.

Beyond the typical morphological abnormalities, the ultrastructural defects observed in the longitudinal sections of the sperm were associated with severe axonemal and periaxonemal defects, which appeared completely disorganized, resulting in aborted flagella or their replacement by a cytoplasmic mass englobing unassembled axonemal components. In *Cfap251^+/+^* mice, the MS was uniformly distributed along the axoneme, whereas *Cfap251^-/-^* mice exhibited disorganized MS structures, mainly characterized by a shortened and thickened appearance (Figure 4A). As illustrated in Figure 4B, a misalignment of TOMM20 signals was observed in *Cfap251^-/-^* mice.

The annulus is a crucial component of the flagellum that connects the MS to the fibrous sheath. By employing immunofluorescence analysis, we observed that SEPT4 exhibited irregular distribution or was absent in sperm from *Cfap251^-/-^* mice (Figure 4C). Statistical analysis revealed significantly elevated proportions of short MS with mislocated annuli in *Cfap251^-/-^* mice (Figure 4D and E).

### Deletion of *Cfap251* causes axonemal defects in sperm flagella and cilia in mice

In *Cfap251*^-/-^ mice, abnormal flagella were observed, and CFAP251 signals were missing in the flagella of elongated spermatids. Previous studies on protist homologs of CFAP251, including those in *Tetrahymena*, *Chlamydomonas*, and *Trypanosoma brucei*, have demonstrated that these proteins are conserved components of the flagellar and ciliary axonemes and are essential for the assembly of RS3. To explore the mechanism by which CAFP251 deficiency leads to MMAF, immunofluorescence analysis was performed to determine the location of CFAP251 among the axonemal components. We utilized sperm from patients carrying pathogenic *LRRC23* mutations and from *Iqub^-/-^* mice as models representing the concurrent absence of RS2/RS3 heads and complete loss of RS1, respectively. Immunofluorescence analysis revealed that CFAP251 expression remained unaffected in both models, suggesting its role as a conserved component of the RS3 stalk. Similarly, LRRC23 expression was unaltered in *Cfap251^-/-^* mice. These results indicate that CFAP251 is an evolutionarily conserved RS protein within the flagella of both humans and mice, localized specifically to the stalk region of RS3, adjacent to the inner dynein arms, and exhibits independent assembly (Supplementary Figure 3).

Further TEM analysis was conducted on sperm from the cauda epididymis of *Cfap251^+/+^* and *Cfap251^-/-^* mice. Flagellar axonemes of *Cfap251^+/+^* mice exhibited a typical “9+2” microtubular arrangement with clearly visible CP, RS, and ODAs. In contrast, sperm from *Cfap251^-/-^* mice exhibited severe abnormalities in all analyzed sections, characterized by CP and RS loss (Figure 4F). Additionally, immunofluorescence was performed to assess the locations and expression levels of ODA-related protein DNAI2, ODF-related protein ODF2, CP-related protein SPAG6, and RS-related proteins RSPH1 and RSPH3 (Figure 4G-K). *Cfap251^-/-^* sperm showed significantly reduced signals for SPAG6, RSPH, RSPH3, and DNALI2 along the flagella. In contrast, ODF2 signals remained unaffected in the midpiece but failed to extend properly to the endpiece. These data further confirmed that defective *CFAP251* gene led to abnormal assembly of flagellar axonemes.

Given the detection of CFAP251 protein in cilia cells, we also examined whether CFAP251 deficiency causes PCD symptoms in males. The ultrastructures of ciliary axonemes showed that the “9+2” microtubule pattern of the cilia was mostly preserved; however, the inner dynein arm structures were significantly reduced or absent (Supplementary Figure 4A-C). Additionally, *Cfap251^-/-^* males developed normally, but Cfap251 expression was absent in tracheal cilia cells (Supplementary Figure 4D and E). Collectively, these findings suggest that CFAP251 deficiency compromises sperm axonemal integrity in mice, manifesting as atypical PCD symptoms.

### CFAP251 interacts with TUBB4B and SLC25A4

**Figure 5:**
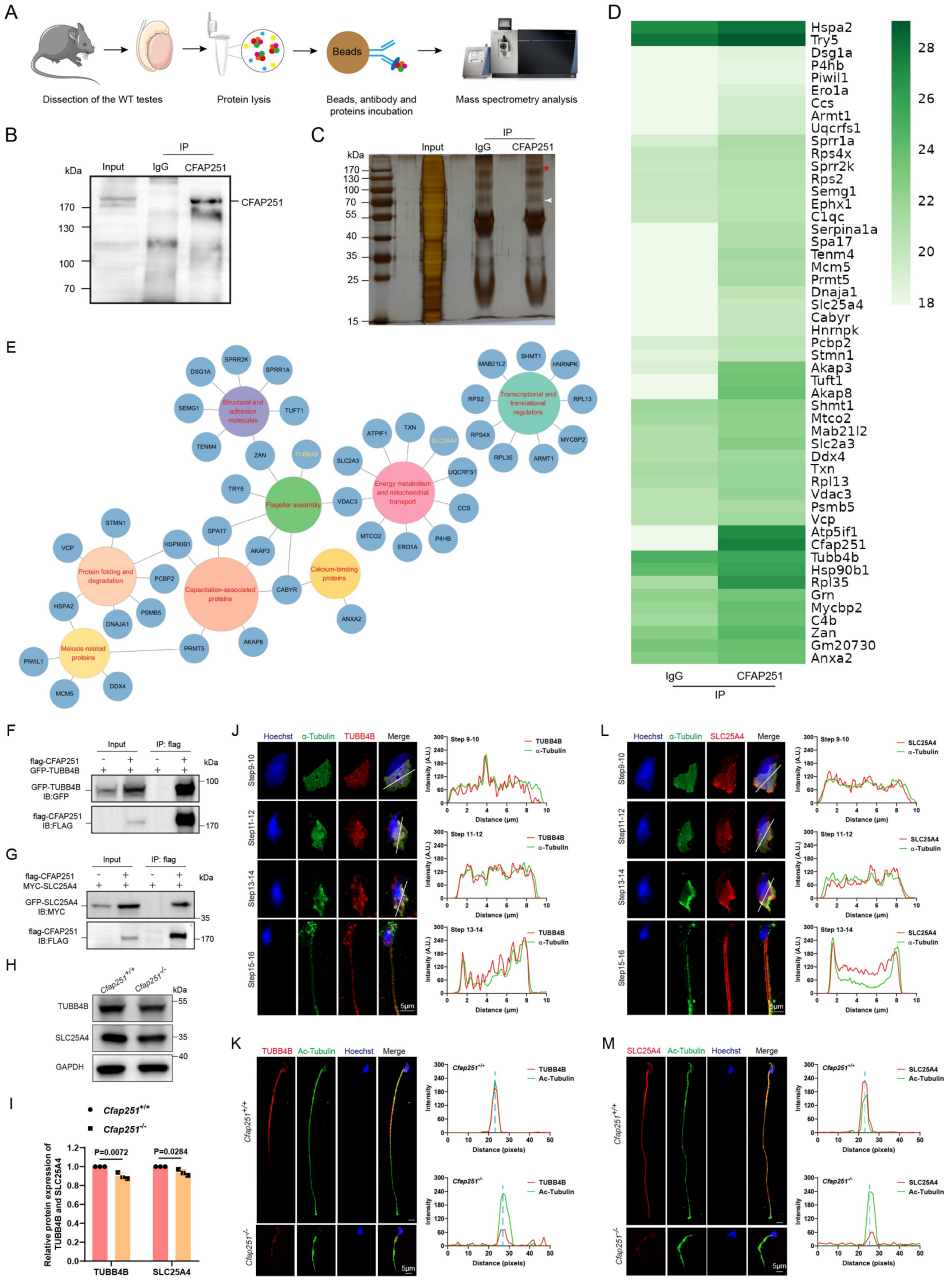
** Identification of CFAP251-interacting proteins and validation of binding partners. (A)** Workflow of CFAP251 immunoprecipitation (IP) and mass spectrometric analysis of wild-type mouse testes. **(B, C)** Immunoblotting (B) and silver staining (C) of protein complexes immunoprecipitated with the CFAP251 antibody compared with the IgG control. **(D)** Heatmap of proteins enriched in the CFAP251 immunoprecipitates. **(E)** Functional clustering of CFAP251-associated proteins based on Gene Ontology annotation. **(F, G)** Co-immunoprecipitation assays confirming interactions between CFAP251 and TUBB4B (F) or SLC25A4 (G). **(H, I)** Immunoblotting (H) and quantification (I) showing altered expression of TUBB4B and SLC25A4 in *Cfap251^-/-^* testes compared to wild-type controls (n = 3 per genotype). GAPDH was used as a loading control. Data are presented as mean ± SD, and statistical test is a two-tailed Student's t-test. p=0.027 for TUBB4B, p=0.0284 for SLC25A4. **(J-M)** Immunofluorescence staining of spermatids with antibodies against TUBB4B (Scale bar, 5 µm) (J, K) and SLC25A4 (L, M), together with acetylated tubulin and Hoechst. Line-scan analyses (right) showing reduced intensity of these axonemal proteins in *Cfap251^-/-^* sperm compared with controls. Scale bar, 5 µm.

**Figure 6:**
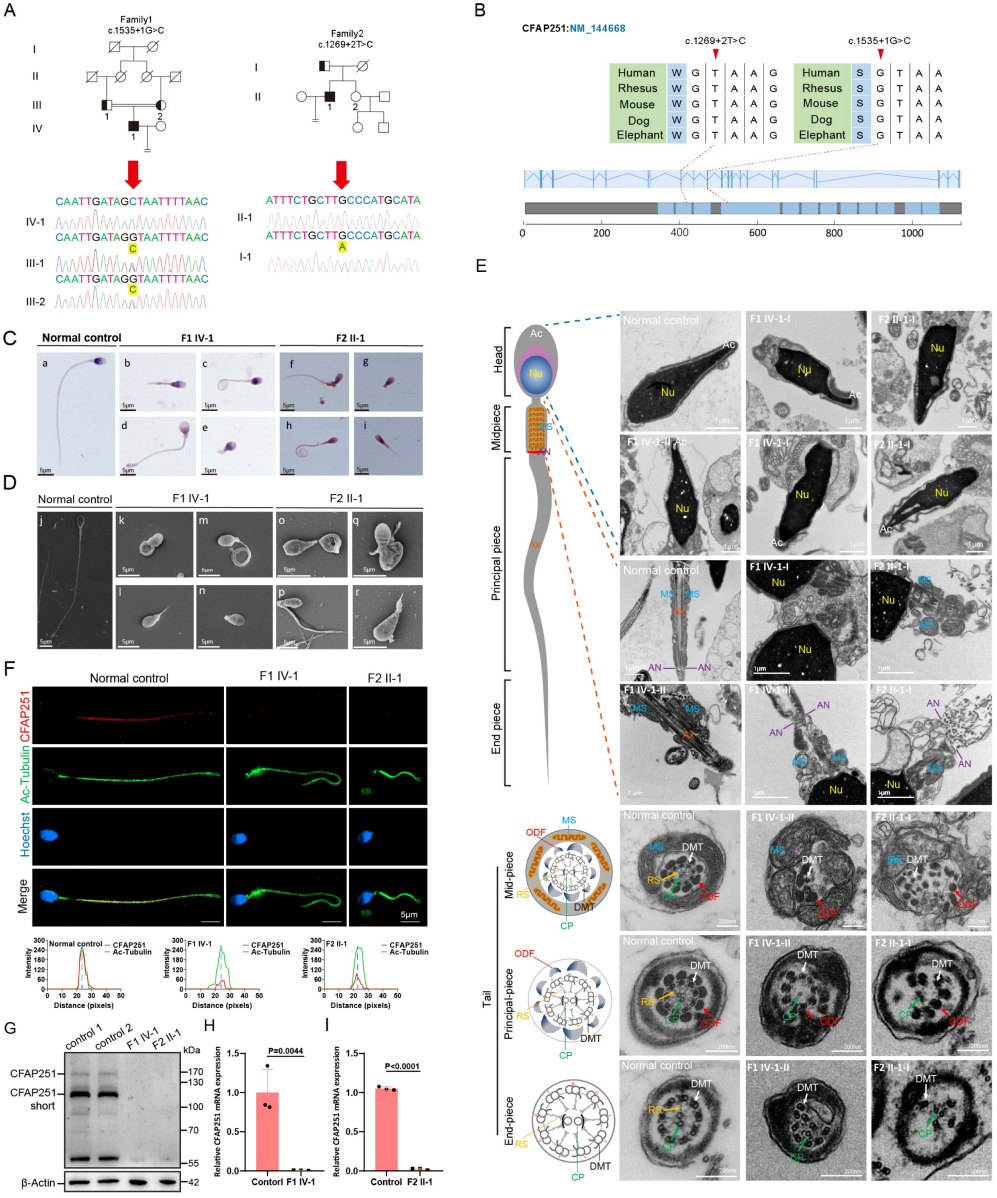
** Pathogenic *CFAP251* variants cause structural and functional defects in human spermatozoa. (A)** Pedigrees of two unrelated families carrying splice-site variants of *CFAP251* (c.1553+1G>C and c.1289+2T>C). Filled symbols denote affected individuals, and arrows indicate probands. Representative Sanger chromatograms confirm the nucleotide changes (yellow arrowheads). **(B)** Schematic representation of the *CFAP251* transcript (NM_144668) showing the positions of the splice-site variants. Sequence alignments demonstrate evolutionary conservation of the affected nucleotides across vertebrate species. **(C)** H&E staining of spermatozoa from patients (F1 IV-1, F2 II-1) and healthy controls showing severe morphological abnormalities of sperm flagella. Scale bar, 5 µm. **(D)** Scanning electron microscopy images showing multiple flagellar defects in patients' sperm, including absent, short, coiled, and irregular flagella. Scale bar, 5 µm. **(E)** TEM images and schematics of the sperm flagella showing axonemal disorganization and accessory structure defects (Ac, acrosome; Nu, nucleus; MS, mitochondrial sheath; AN, annulus; Ax, axoneme; DMT, outer doublet microtubules; CP, central pair; RS, radial spokes; ODF, outer dense fibers). Scale bar: 1 µm, 200 nm. **(F)** Immunofluorescence staining for CFAP251 (red) and acetylated α-tubulin (green) in sperm flagella, showing loss of CFAP251 signal in affected individuals. Line-scan intensity profiles indicate the absence of CFAP251 localization. Scale bar, 5 µm. **(G)** Immunoblot analysis of CFAP251 in sperm protein extracts from control and affected individuals. Relative quantification demonstrates a significant reduction or absence of the CFAP251 protein in patients. **(H, I)** qRT-PCR analyses show significantly decreased *CFAP251* mRNA levels in patient sperm compared with those in controls (n = 3 per group). Data are presented as mean ± SD, and statistical test is a two-tailed Student's t-test. p= 0.0044 for F1 IV-1 and p<0.0001 for F2 II-1.

To elucidate the molecular mechanism of CFAP251, we conducted Co-IP followed by mass spectrometry of testicular lysates from adult *Cfap251^+/+^* mice (Figure 5A-C). Gene ontology analysis revealed that the potential interactors were mainly enriched in flagellar assembly, mitochondrial transport, and capacitation-associated proteins (Figure 6D and E). Among these proteins, TUBB4B, SLC25A4, CABYR, and AKAP3 were annotated as closely related to the defects observed in *Cfpa251^-/-^* mice. This piqued our interest to explore these proteins in detail. Co-IP assays demonstrated strong interactions between TUBB4B, SLC25A4, and CFAP251 *in vitro* (Figure 6F and G), whereas only weak interactions were observed between CABYR and AKAP3 (Supplementary Figure 5). To evaluate the effects of *CFAP251* deletion on these proteins during spermiogenesis, we examined their abundance in mouse testis lysates. The levels of TUBB4B and SLC25A4 were reduced in *Cfap251^-/-^* testes, whereas CABYR and AKAP3 levels were unaffected (Figure 6H and I). Given their weak interactions with CFAP251 and unaltered expression in *Cfap251*^-/-^ testes, CABYR and AKAP3 proteins were not pursued further, allowing us to focus the subsequent investigations on the stronger candidate interactors TUBB4B and SLC25A4. Immunofluorescence staining for TUBB4B and SLC25A4 on testicular cell smears further revealed extensive colocalization of both proteins with tubulin in elongating spermatids at steps 9-16. In mature spermatozoa, TUBB4B and SLC25A4 labeling was observed along the entire flagellum, with TUBB4B signal enriched in the principal piece and SLC25A4 concentrated in the midpiece (Figures 5J and L). Given that CFAP251 deficiency caused diminished immunofluorescence signals for TUBB4B and SLC25A4 in the sperm flagella of mice (Figures 5K and M), we propose that the interaction between CFAP251, TUBB4B, and SLC25A4 is required for flagellar development during spermiogenesis. Protein-protein complexes structure modeling and binding affinity predictions for the CFAP251-TUBB4B and CFAP251-SLC25A4 complexes support this hypothesis, indicating that these proteins can efficiently interact (Figures S6). The predicted binding models reveal that the intermolecular interfaces between CFAP251, TUBB4B, and SLC25A4 are stabilized by a network of hydrogen bonds and hydrophobic interactions, which together contribute to the formation of thermodynamically favorable protein-protein complexes (Figures S6). These findings underscore the critical role of the interactions between CFAP251 and TUBB4B, as well as CFAP251 and SLC25A4, in flagellar morphogenesis and sperm function.

### Two novel homozygous splicing mutations in *CFAP251* were identified in the probands with MMAF

To further validate these findings in humans and expand the mutation spectrum, a cohort of patients with idiopathic severe teratoasthenospermia was recruited during the course of this study. Using whole-exon sequencing, two homozygous splicing mutations in *CFAP251* were identified c.1535+1G>C and c.1269+2T>C in two unrelated probands (F1 IV-1 and F2 II-1). These mutations were verified by Sanger sequencing, and their parents were heterozygous carriers (Figure 6A and B). Both mutations were absent in the 1000G, ExAC, and gnomAD databases. Routine semen analysis of the two probands revealed severe teratoasthenospermia with complete loss of sperm motility (Table 1). Sperm morphology analysis of the two probands revealed typical MMAF characteristics (Figure 6C). SEM further confirmed the MMAF phenotype in most spermatozoa from both probands (Figure 6D). Statistical analysis indicated that flagellar abnormalities were predominantly characterized by coiled and short tails. Detailed information is presented in Supplementary Table 5. Furthermore, additional history taking revealed that the proband (F1 IV-1) frequently experienced tinnitus and a seasonal cough. Subsequent chest CT imaging demonstrated coarsened lung markings and a solitary pulmonary bulla, findings associated with chronic bronchitic inflammation (Supplementary Figure 7). Although these clinical manifestations and radiological findings are insufficient to confirm a definitive diagnosis of PCD, they serve as strong suggestive evidence for the disorder.

### Sperm ultrastructure analysis of the probands carrying *CFAP251* homozygous splicing mutations

To further examine the ultrastructure, TEM was performed on spermatozoa obtained from the normal male controls and the two probands. Longitudinal sections showed severe acrosomal and periaxonemal defects affecting the fibrous sheath and MS, resulting in detached acrosomes or aborted flagella (Figure 6E). Immunofluorescence assays revealed accumulation of the TOMM20 signal, accompanied by loss or aberrant localization of the SEPT4 signal in sperm flagella obtained from both probands (Supplementary Figure 8A and B). Western blot analysis revealed a significant reduction in mitochondrial content in the spermatozoa of *Cfap251^-/-^* mice compared to that of *Cfap251^+/+^* mice, which was consistent with the abnormalities in the MS and annulus observed in over 80% of the sperm (Supplementary Figure 8C-E). Cross-sections showed the typical "9+2" axonemal structure along the flagella in control spermatozoa. However, various axonemal anomalies were observed in the spermatozoa from F1 IV-1 and F2 II-1, including disordered ODFs and DMTs lacking RS and CP (Figure 6E). Consistent with the observations in mice, the signals of several major structural markers were also significantly downregulated in the sperm flagella of the probands (Supplementary Figure 8F-J). Furthermore, CFAP251 was detected along the length of the flagella in spermatozoa from the control individuals but was absent in those of F1 IV-1 and F2 II-1, confirming a complete loss of CFAP251 (Figure 6F). Subsequent western blotting and qRT-PCR detected significantly decreased expression levels of CFAP251 protein and mRNA in F1 IV-1 and F2 II-1 compared with controls (Figure 6G-I).

To evaluate the functional mechanism by which CFAP251 deficiency affects flagellar structure in humans, we examined the expression of TUBB4B and SLC25A4 proteins in spermatozoa using immunofluorescence. The results revealed a significant downregulation of the colocalization signals for both TUBB4B and SLC25A4 in the sperm flagellum (Supplementary Figure 9A and B). Given that CFAP251 deficiency causes defects in dynein arm assembly within the ciliary axoneme of mice, we speculated that this effect might be conserved across mammals. Owing to the invasive nature of ciliary biopsy, a chest CT scan was performed for proband F1, IV-1, revealing slightly increased lung markings and the presence of pulmonary bullae in the right lung. These findings suggest that CFAP251-deficient individuals may be susceptible to PCD because of impaired ciliary motility.

### Successful ICSI outcomes in infertile men with *CFAP251* pathogenic variants and in *Cfap251^-/-^* mice

Since the patients with *CFAP251* mutation and *Cfap251^-/-^* mice have similar deficiencies, we evaluated whether patients with asthenoteratozoospermia caused by *CFAP251* variants could be treated with assisted reproductive technology. Specifically, spermatozoa from *Cfap251^+/+^* and *Cfap251^-/-^* mice were used for ICSI. The results showed that the rates of two-cell embryo formation and blastocyst development in the *Cfap251^-/-^* group were comparable to those in the *Cfap251^+/+^* group (Supplementary Table 6). We further reviewed the pregnancy outcomes of the couples in this study and two cases from our previous report. All four couples (F1 IV-1, F2 II-1, M1 IV-1, and M4 II-1) achieved clinical pregnancy after ICSI treatment and subsequently delivered healthy infants. Detailed ICSI outcomes for patients carrying *CFAP251* mutations are summarized in Table 1.

## Discussion

In this study, we generated *Cfap251* knockout mice as an animal model, which successfully replicated the MMAF phenotype observed in humans, thereby confirming the pathogenic role of *CFAP251* mutations in asthenoteratozoospermia. Using testicular Co-IP, mass spectrometry proteomics, structural modeling, and binding affinity predictions, we found that CFAP251 interacts with TUBB4B and SLC25A4 and serves as part of a structural scaffold required to facilitate the stable incorporation of TUBB4B and SLC25A4 at the sperm flagella, highlighting the crucial role of CFAP251 in sperm flagellar development. Additionally, we identified two novel homozygous splicing mutations in *CFAP251* that cause the loss of CFAP251 protein in patients with MMAF. Spermatozoa from these patients exhibited abnormal morphology and axonemal defects, including a high percentage of impaired IDAs and loss of CP. Notably, CFAP251 deficiency manifests as atypical PCD symptoms in humans and mice, underscoring the role of CFAP251 in ciliary motility. We also demonstrated that ICSI could overcome infertility in the affected probands, as evidenced by the successful delivery of healthy offspring.

The *CFAP251* gene is located on chromosome 12q24.31, and the protein encoded by this gene belongs to the WD repeat-containing family, which functions in the formation of protein-protein complexes across diverse biological pathways. Unlike many CFAP family members that utilize domains such as WD repeats and coiled-coil domains for specific binding interactions, CFAP251 is characterized by a notably long intrinsically disordered N-terminal region, positioning it as a prominent scaffold protein within the family. According to the STRING database (https://cn.string-db.org), CFAP251 interacts with MAATS1, CFAP43, CFAP61, CFAP69, CFAP70, DNAH1, QRICH2, TTC29, ARMC2, and FSIP2. Defects in these genes have been associated with asthenoteratozoospermia, accompanied by diminished levels or the absence of axoneme-associated proteins within the sperm flagella. Specifically, pathogenic variants of *CFAP43, DNAH1*, and *ARMC2* are associated with the multisystem phenotypes of PCD 17-19. These results provide additional evidence supporting the critical involvement of CFAP251 in axonemal assembly across both flagella and cilia. We detected weak interactions between CFAP251 and CABYR/AKAP3 through supplementary proteomic analyses; however, due to the lack of significant expression differences, we chose to further investigate the mechanism involving TUBB4B. Studies on patients with PCD and mouse models have demonstrated that mutations in *TUBB4B* disrupt ciliogenesis, resulting in reduced cilium number and length, as well as axonemal structural abnormalities 20, 21. Although the loss of CFAP251 in the respiratory cilia of *Cfap251^-/-^* mice did not result in overt defects in ciliary length or density, TEM revealed a frequent absence of inner dynein arms within the axoneme. Notably, one patient carrying biallelic CFAP251 mutations showed PCD-like symptoms, including tinnitus, seasonal cough, and radiological findings of coarsened lung markings and a solitary pulmonary bulla associated with chronic bronchitic inflammation. These clinical manifestations, along with the radiological findings, highlight the underlying risk of male infertility and PCD-like ciliary defects. Although these findings are insufficient to confirm a definitive diagnosis of PCD, they serve as strong suggestive evidence for the disorder. Moreover, CFAP251 is a prerequisite for the proper assembly of TUBB4B onto sperm flagella in both humans and mice, which further supports our hypothesis.

Deficiency in CFAP251 resulted in sperm ultrastructural defects, such as CP loss and impaired RSs, indicating the essential role of CFAP251 in maintaining axonemal integrity and arrangement. Our findings revealed decreased or completely absent expression of CP-related protein SPAG6, RS-related proteins RSPH1 and RSPH3, ODA-related protein DNAI2, and ODF-related protein ODF2 in the sperm of *Cfap251^-/-^* mice compared with that of controls. Similar findings have been documented in multiple reports, revealing pronounced abnormalities in axonemes and their adjacent structures among patients with *CFAP251* variants 22-24. These intricate interactions underscore the delicate balance within the sperm flagellar proteome. The localization of CFAP251 undergoes dynamic changes during spermiogenesis, particularly during acrosomal migration (steps 9-13) and chromatin condensation (steps 14-16). Anatomically, CFAP251 is initially expressed in the manchette structure of the elongating spermatid and later becomes enriched in the tail of the elongated spermatid. The manchette and sperm tail axonemes are two microtubular platforms that transport proteins to the developing head and tail. The manchette is a transient skirt-like structure that functions as a scaffold for morphological remodeling, contributing to the formation of the sperm head, neck, and tail 25. We found that CFAP251 deficiency resulted in an abnormal manchette structure and compromised sperm head and tail morphologies.

Additionally, previous studies have documented alterations in the MS ultrastructure in individuals harboring *CFAP251* variants 26. Disturbances in the MS have also been observed in patients with *CFAP251* biallelic mutations and knockout mice. Consistently, our investigation revealed that CFAP251 interacts with SLC25A4, a component of the mitochondrial permeability transition pores 27, and Protein-protein Complexes Structure Modeling and Binding Affinity Prediction assays further confirmed their potential binding interface at the molecular level. And in *Cfap251^-/-^* testes, SLC25A4 protein expression was significantly reduced. These observations imply that CFAP251 not only contributes to the assembly of the axonemal structure but also exerts a substantial influence on the proper distribution and functioning of MS.

In current clinical practice, ICSI has emerged as a key intervention for patients with MMAF. However, ICSI may not be suitable for all MMAF cases. Patients carrying *CFAP65* mutations have poor ICSI outcomes 28, whereas those with *CFAP69* 29*, CFAP47* 30*,* or *CFAP52* 31 mutations have achieved favorable clinical pregnancies. Regarding *CFAP251*, only one case of clinical pregnancy was reported in 2022. Our study demonstrated successful ICSI outcomes in *Cfap251^-/-^* mice, suggesting that ICSI is an appropriate intervention for infertile patients with *CFAP251* mutations. Furthermore, we identified two novel homozygous splicing variants of *CFAP251* and reported favorable live birth outcomes following ICSI in four probands carrying *CFAP251* mutations. Collectively, this evidence provides a foundation for the clinical management of patients harboring such mutations.

It is important to acknowledge certain limitations of the present study. we were unable to obtain nasal nitric oxide (nNO) measurements or ciliary biopsy results due to clinical testing constraints, precluding definitive confirmation of the link to PCD. Another notable limitation is that while ciliary dynein arm defects are clearly demonstrated in the mouse model (Supplementary Figure 4), human ciliary ultrastructure data could not be generated owing to the unavailability of patient ciliary biopsy samples, meaning the PCD-like phenotype in humans was inferred solely from symptoms and chest CT findings rather than direct morphological evidence. Furthermore, although ICSI has shown promising outcomes for *CFAP251* mutation carriers, it is important to consider the potential risk of passing on genetic infertility traits to offspring.

In conclusion, our study functionally and mechanistically elucidated a special and poorly characterized protein, CFAP251, in sperm flagellar biogenesis and its distinct function in respiratory cilia. Combined with our previous findings, we confirm that patients with MMAF caused by biallelic *CFAP251* variants can achieve successful delivery of healthy offspring. These findings have significant implications for the diagnosis and treatment of male infertility associated with *CFAP251* variants.

## Supplementary Material

Supplementary figures and tables.

## Figures and Tables

**Table 1:** ICSI outcomes of individuals with *CFAP251* variants

Patients	F1 IV-1	F2 II-1	M1 IV-1	M4 II-1
Male age (years)	26	24	34	30
Female age (years)	25	24	36	29
No. of ICSI cycles	1	1	1	1
No. of oocytes injected	4	23	10	15
Fertilization rate (%)	75(3/4)	74(17/23)	90(9/10)	93.3(14/15)
Cleavage rate (%)	100(3/3)	100(17/17)	100(9/9)	100(14/14)
8 cells embryo development rate (%)	100(3/3)	100(17/17)	55.6(5/9)	42.9(6/14)
Blastocyst formation rate (%)	67(2/3)	76(13/17)	55.6(5/9)	28.6(4/14)
No. of frozen-thawed embryos transfer cycles	2	1	1	1
Number of embryos transferred	1	1	2	1
Implantation rate (%)	50(1/2)	100(1/1)	50(1/2)	100(1/1)
Clinical pregnancy	Y	Y	Y	Y
Live birth	Y	Y	Y	Y

Annotations: ICSI, intracytoplasmic sperm injection; Y, yes; N, no. The M1 IV-1 and M4 II-1 are reported in our previous study.

## Data Availability

The identified variants are publicly available in the Leiden Open Variation Database (LOVD). URL: https://databases.lovd.nl/shared/variants/0001059339#00025372, https://databases.lovd.nl/shared/variants/0001059551#00025372.
