# Supplementary material for: CFAP251 Deficiency Induces Male Infertility and PCD-like Ciliary Defects by Disrupting TUBB4B and SLC25A4 Recruitment in Humans and Mice
Source: Int J Biol Sci. 2026 Feb 26;22(6):2970–85. doi: 10.7150/ijbs.128822 (PMC13050454; doi:10.7150/ijbs.128822)
Supplement: Supplementary file 1 — Supplementary figures and tables. [file ijbsv22p2970s1.pdf]

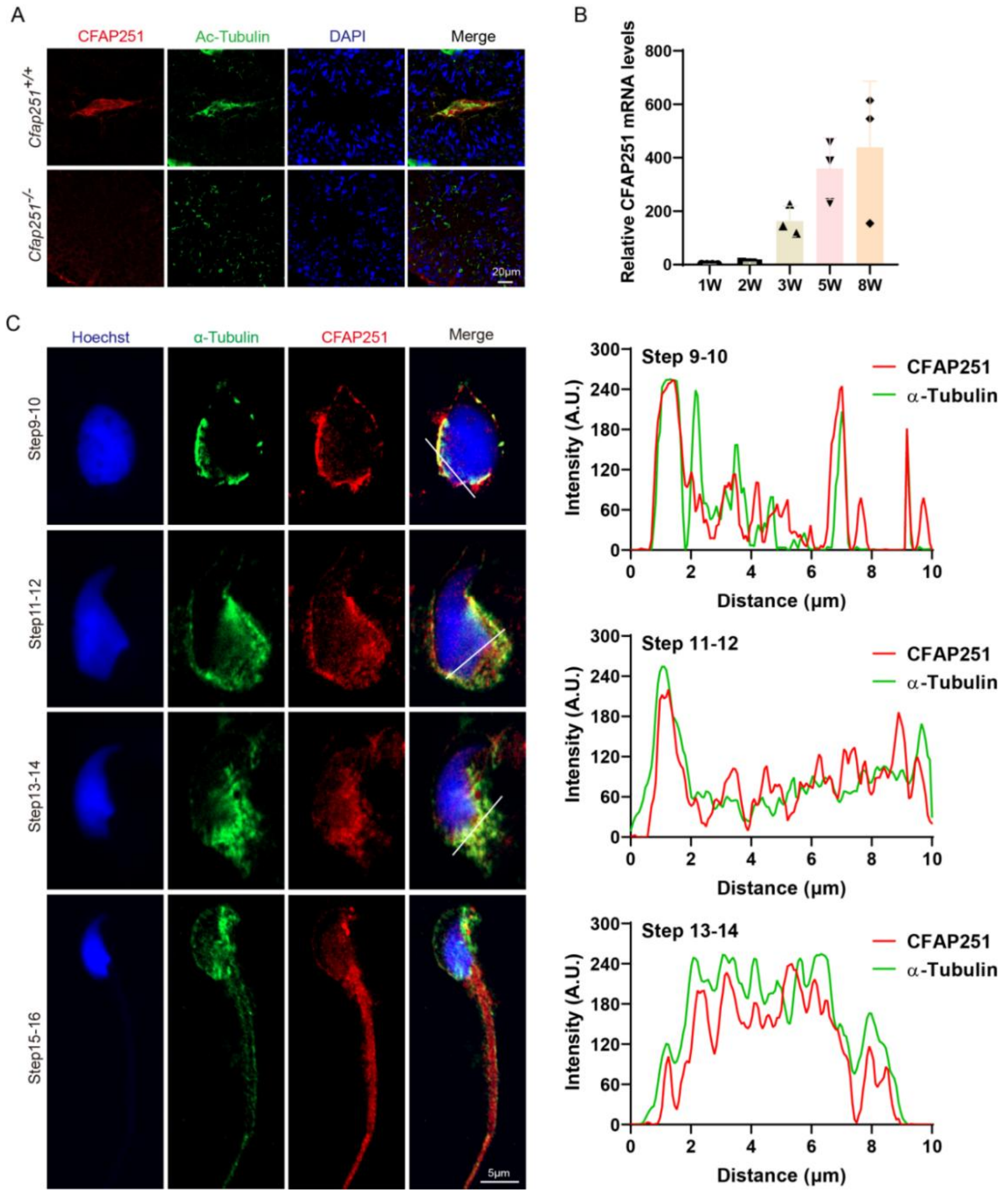

Figure S1. ***CFAP251*** **colocalizes with tubulin in developing sperm flagella and manchette.**

(A) Immunofluorescence staining of testicular sections from *Cfap251*$^{+/+}$ and *Cfap251*$^{-/-}$ mice. CFAP251 (red) colocalizes with acetylated-tubulin (green) in the flagella of spermatozoa within the seminiferous tubules, while DAPI (blue) marks nuclei. Signal for CFAP251 is absent in *Cfap251*$^{-/-}$ mice. Scale bar, 20μm.(B) Relative mRNA expression levels of Cfap251 in testes at different postnatal ages, showing progressive upregulation during spermatogenesis. Gapdh was used for norminalization. Data were presented as mean±SEM, n=3 (three biological replicates) (C) Immunofluorescence staining of developing spermatids at steps 9–16, showing colocalization of CFAP251 (red) with α-tubulin (green) at the manchette. Hoechst (blue) marks nuclei. Line-scan analysis (right) demonstrates overlapping fluorescence intensity profiles of *CFAP251* and α-tubulin across the manchette. Scale bar, 5μm.

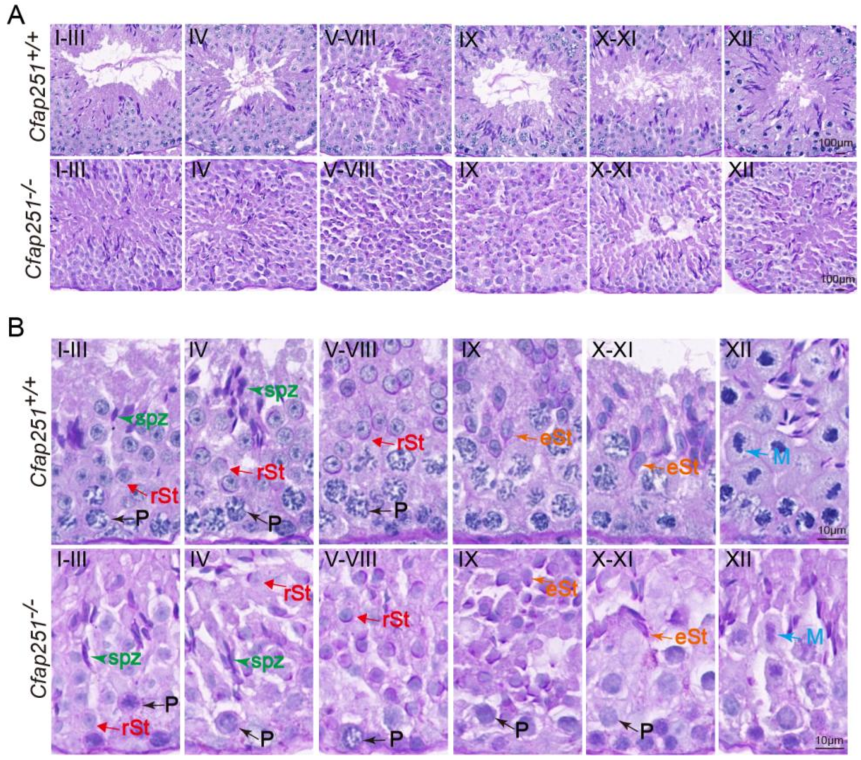

Figure S2. CFAP251 deficiency causes flagellar shortening and sperm head malformations.

(A) Periodic acid–Schiff (PAS) staining of seminiferous tubules at different stages (I–XII) from *Cfap251*$^{+/+}$ and *Cfap251*$^{-/-}$ testes. In *Cfap251*$^{-/-}$ mice, sperm flagella are consistently shorter and abnormally coiled across multiple stages compared with controls. Scale bar, 100µm. (B) Higher magnification of seminiferous epithelium showing spermatids at representative stages. *Cfap251*$^{-/-}$ testes exhibit aberrant head morphology in elongating and late spermatids. Abbreviations: **spz**, spermatozoa; **rSt**, round spermatids; **P**, pachytene spermatocytes; **eSt**, elongated spermatids; **M**, mature spermatozoa. Scale bar, 10µm.

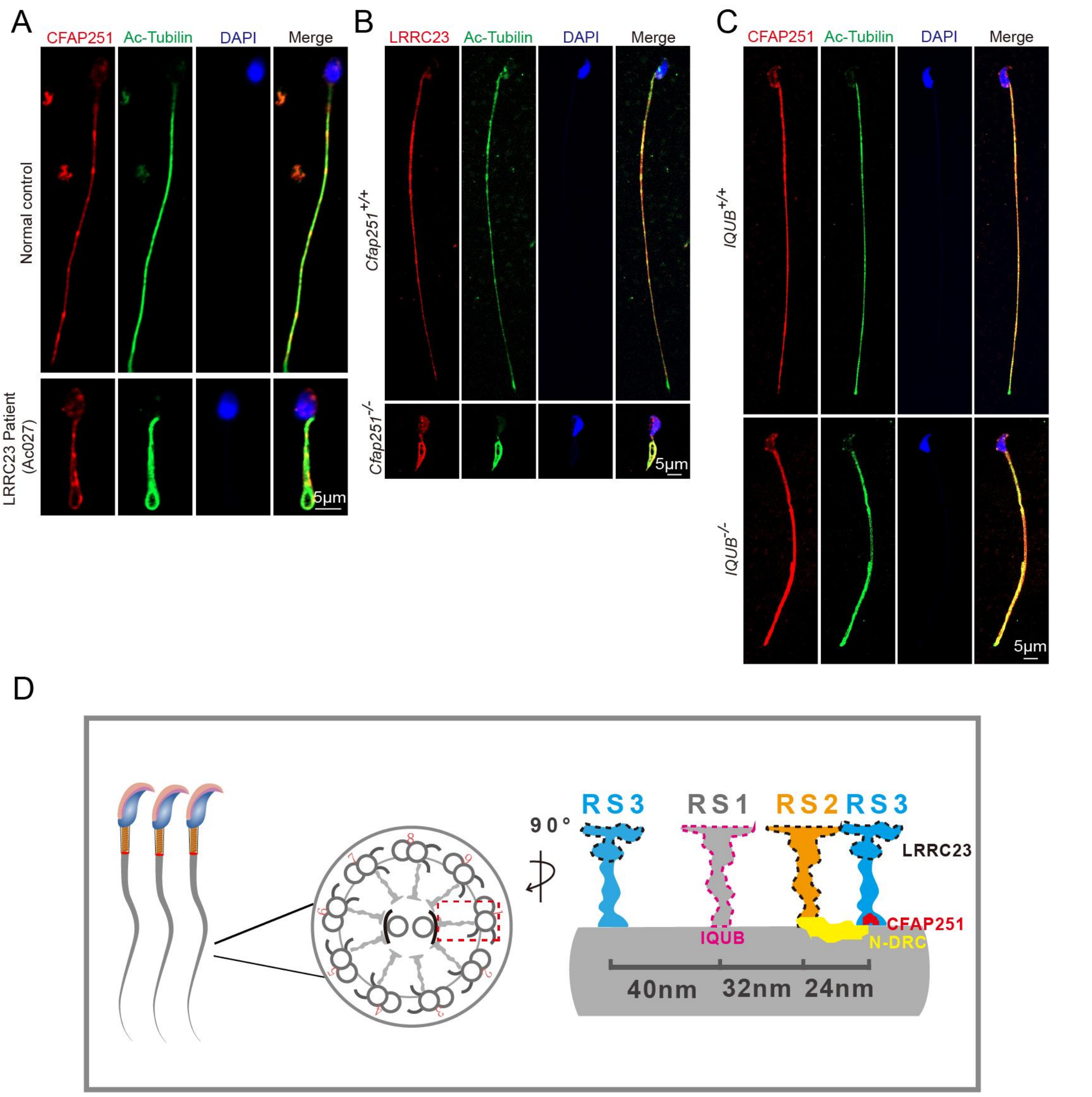

Figure S3. ***CFAP251*** **is not localized to the RS1 or RS2 subunits, nor to the upper region of the RS3 subunit in sperm flagella.**

(A) *CFAP251* localization (red) in spermatozoa from an *LRRC23*-mutant patient (Ac027,(c.376C>T:p. Arg126X)) shows no significant difference compared with normal control; acetylated α-tubulin (green) and DAPI (blue) mark axoneme and nuclei, respectively. Scale bar, 5μm. (B) *LRRC23* localization (red) in *Cfap251*$^{-/-}$ mouse sperm flagella shows no significant difference compared with *Cfap251*$^{+/+}$. Scale bar, 5μm. (C) *CFAP251* localization (red) in *Iqub*$^{-/-}$ mouse sperm shows no significant difference compared with wild-type (Iqub^{+}/$^{+}$). Scale bar, 5μm. (D) Schematic of the radial spoke (RS) complex indicating IQUB at the RS1 subunit and LRRC23 at the upper regions of the RS2 and RS3 subunits. Together, these data indicate that *CFAP251* is not localized to the RS1 or RS2 subunits, nor to the upper region of the RS3 subunit.

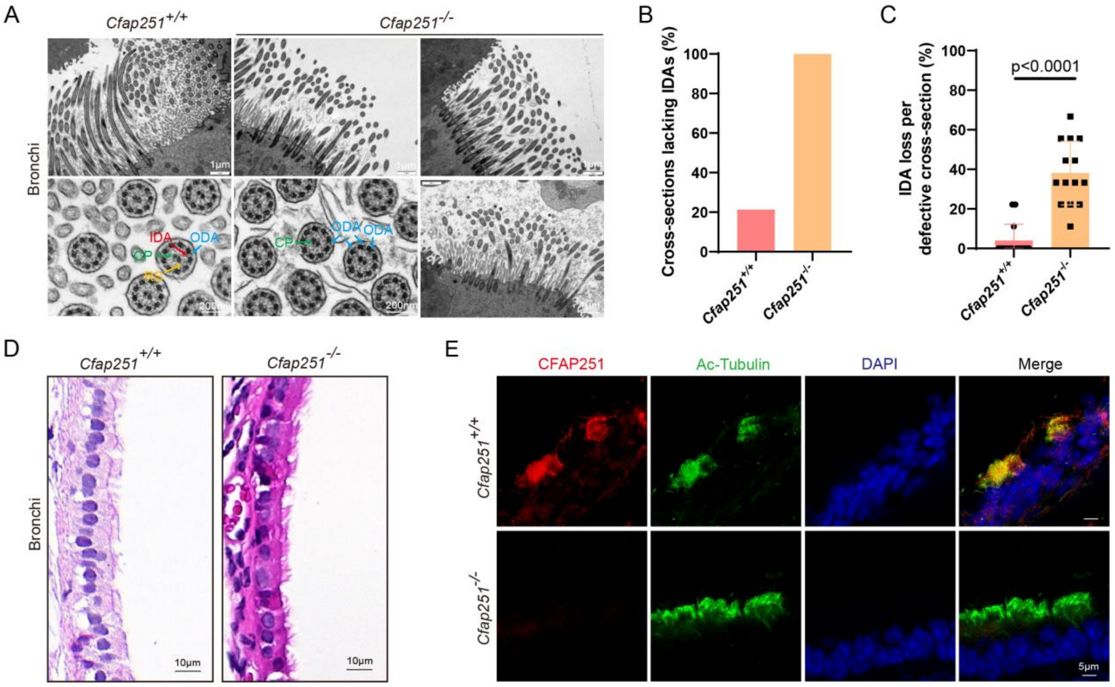

Figure S4. Dynein arm loss and ciliary defects in *Cfap251* knockout mice.

(A) TEM of bronchial cilia from *Cfap251*$^{+/+}$ and *Cfap251*$^{-/-}$ mice showing severe loss of dynein arms in mutants. Insets indicate outer dynein arms (ODA, blue), inner dynein arms (IDA, red), radial spokes (RS, yellow), and central pair (CP, green). Scale bar, 1μm, 200nm. (B) Proportion of bronchial ciliary cross-sections lacking IDAs: 3 out of 14 *Cfap251*$^{+/+}$ cross-sections and all 14 *Cfap251*$^{-/-}$ cross-sections exhibited IDA deficiency (n = 14 cross-sections per genotype). (C) Proportion of IDA loss per defective cross-section, calculated as the number of missing IDAs divided by the total of 9 IDAs per cross-section (n = 14 cross-sections per genotype). Data are presented as mean ± SD, and statistical test is a two-tailed Student's t-test.$p < 0.0001$. (D) H&E staining showing disorganized bronchial cilia in *Cfap251*$^{-/-}$ mice. Scale bar, 10μm. (E) Immunofluorescence staining of bronchial cilia showing CFAP251 (red) in wild type but absent in mutants; Ac-tubulin (green) marks axonemes and DAPI (blue) labels. Scale bar, 5 μm.

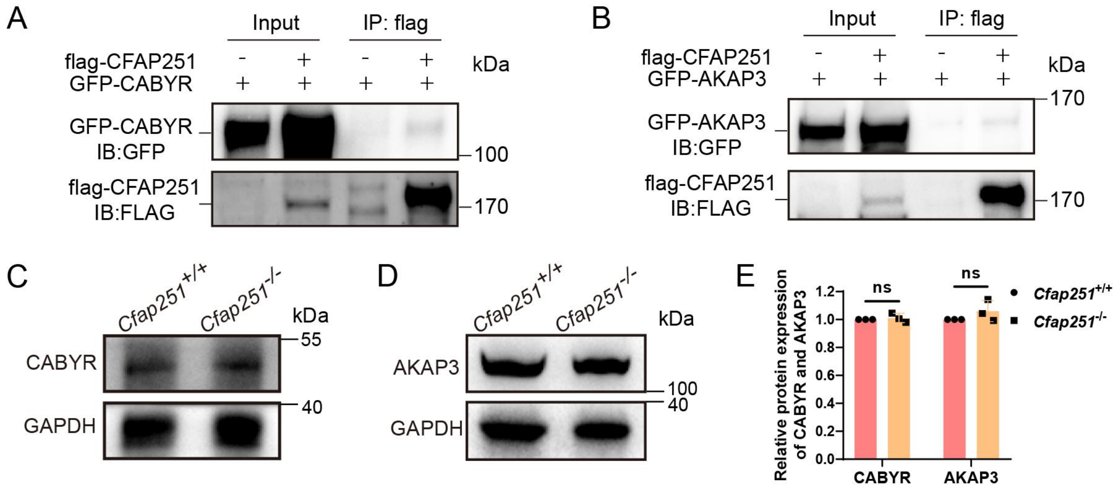

Figure S5. Weak interactions of *CFAP251* with *CABYR* and *AKAP3* and their expression patterns in *Cfap251* knockout mice.

(A, B) Co-immunoprecipitation assays in HEK293T cells showing that CFAP251 weakly interacts with CABYR (A) and AKAP3 (B). FLAG-CFAP251 was immunoprecipitated, and GFP-tagged CABYR or AKAP3 were detected by immunoblotting. (C–E) Immunoblot analysis and quantification of CABYR (C) and AKAP3 (D) expression in testes from wild-type (*Cfap251*$^{+/+}$) and knockout (*Cfap251*$^{-/-}$) mice, showing no significant differences (E). GAPDH was used as a loading control (n = 3 per genotype). Data are presented as mean ± SD, and statistical test is a two-tailed Student's t-test. ns, not significant.

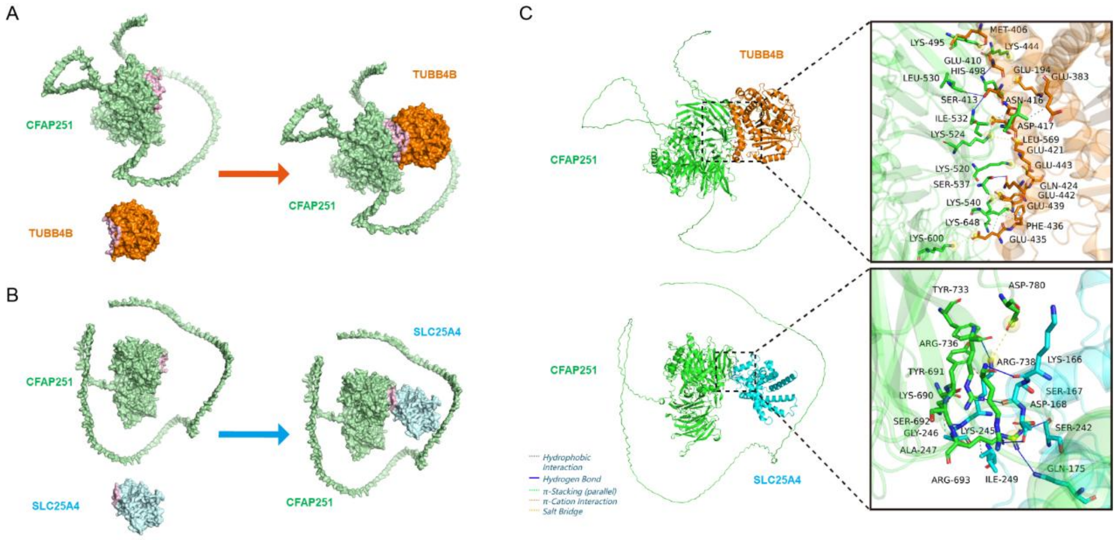

Figure S6. Molecular interaction profiles of CFAP251 with TUBB4B and SLC25A4

(A, B) Surface representations of the binding of CFAP251 (green surface) with TUBB4B (orange surface, A) and SLC25A4 (blue surface, B). The pink-highlighted regions indicate the protein－protein interaction interfaces. Binding affinities, calculated using PRODIGY, were -10.8 kcal/mol for the CFAP251-TUBB4B complex and -7.7 kcal/mol for the CFAP251-SLC25A4 complex. More negative values represent higher thermodynamic stability. (C) 3D interaction diagrams illustrating intermolecular forces at the binding interfaces. CFAP251 is shown in green cartoon representation, TUBB4B in orange, and SLC25A4 in blue. The CFAP251-TUBB4B interface is stabilized by 5 hydrophobic interactions (grey dashed lines), 6 hydrogen bonds (blue solid lines), 9 salt bridges (yellow dashed lines; yellow spheres indicate charge centers), and 1 π-cation interaction (orange dashed lines). No π-π stacking interactions were detected. The CFAP251-SLC25A4 interface consists of 3 hydrophobic interactions, 7 hydrogen bonds, and 2 salt bridges, with no π-π stacking or π-cation interactions observed. Note: Hydrophobic interactions (grey dashed lines), hydrogen bonds (blue solid lines), π-π stacking (green dashed lines), π-cation interactions (orange dashed lines), and salt bridges (yellow dashed lines). Yellow spheres represent charge centers.

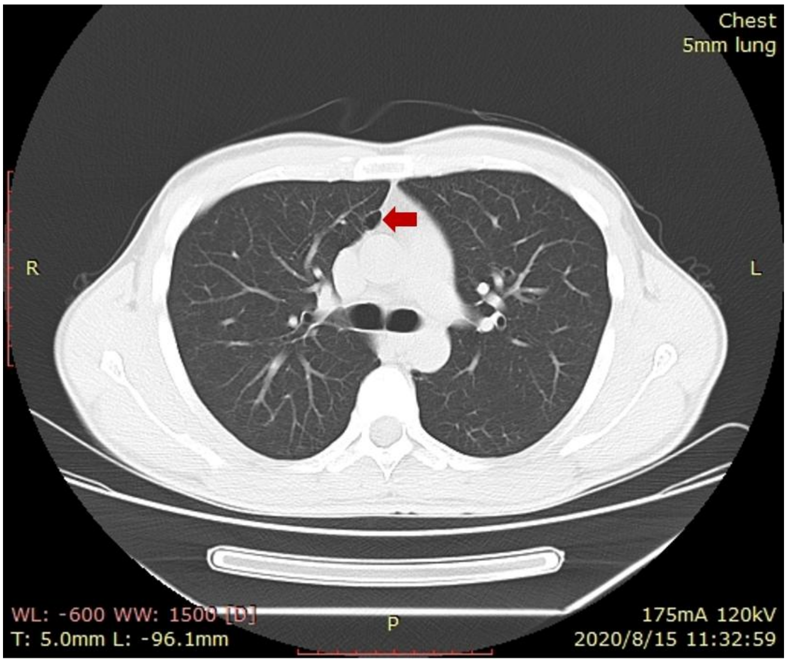

Figure S7. Axial chest CT image (lung window).

A red arrow identifies a pulmonary bulla in the right lower lobe. The surrounding lung parenchyma demonstrates diffusely coarsened interstitial markings, suggestive of underlying chronic bronchitic or fibrotic changes.

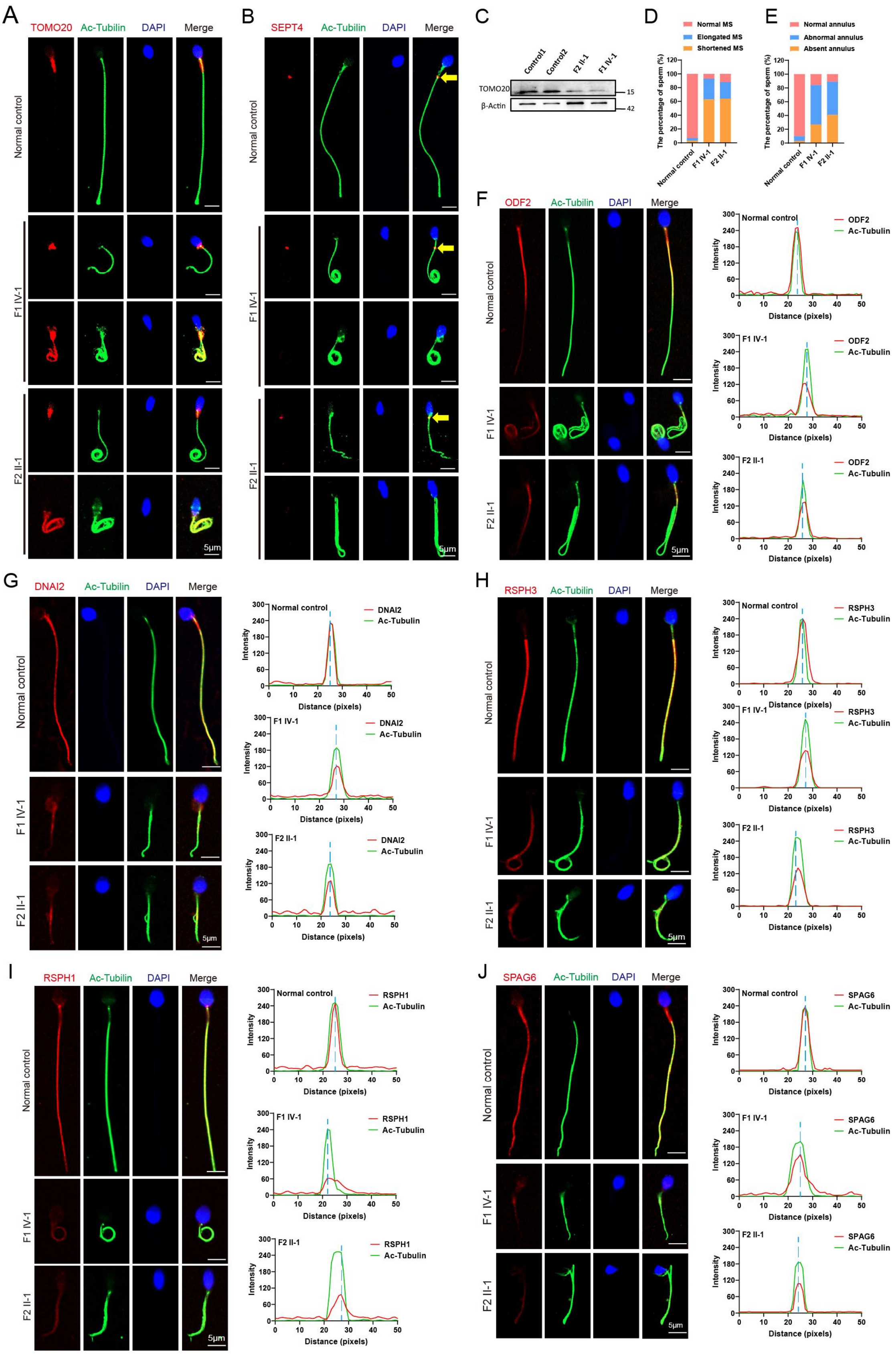

Figure S8. Defective assembly of mitochondrial sheath, annulus, and axonemal components in spermatozoa from affected individuals

(A) Immunofluorescence staining of TOMM20 (red) shows altered mitochondrial

sheath morphology in spermatozoa from affected individuals, with elongated and shortened mitochondria compared with normal control. Acetylated-tubulin (green) marks the axoneme, and DAPI (blue) stains nuclei. Scale bar, 5μm. (B) Immunostaining of SEPT4 (red), a marker of the sperm annulus, reveals either mislocalization or complete absence of the annulus in affected individuals. Scale bar, 5μm. (C) Immunoblot analysis of TOMM20 expression in spermatozoa from control and affected individuals, with β-actin as loading control. (D, E) Enumeration analysis of fluorescence signals from A and B: the fraction of spermatozoa with elongated or shortened MS (D) and with abnormal or absent annulus phenotypes (E) was determined by counting spermatozoa (n = 100 per group), with fractions calculated as spermatozoa of each type divided by the total count, revealing a higher proportion of these phenotypes in affected individuals. (F–J) Immunofluorescence and line-scan analysis of CDF2 (F), DNAI2 (G), RSPH3 (H), RSPH1 (I), and SPAG6 (J) (all red) show reduced expression levels and disrupted distribution along the flagella of affected spermatozoa compared with controls. Acetylated-tubulin (green) marks the axoneme, and DAPI (blue) stains nuclei. Line-scan profiles on the right confirm decreased fluorescence intensity of these proteins in affected individuals, indicating reduced expression. Scale bar, 5 μm.

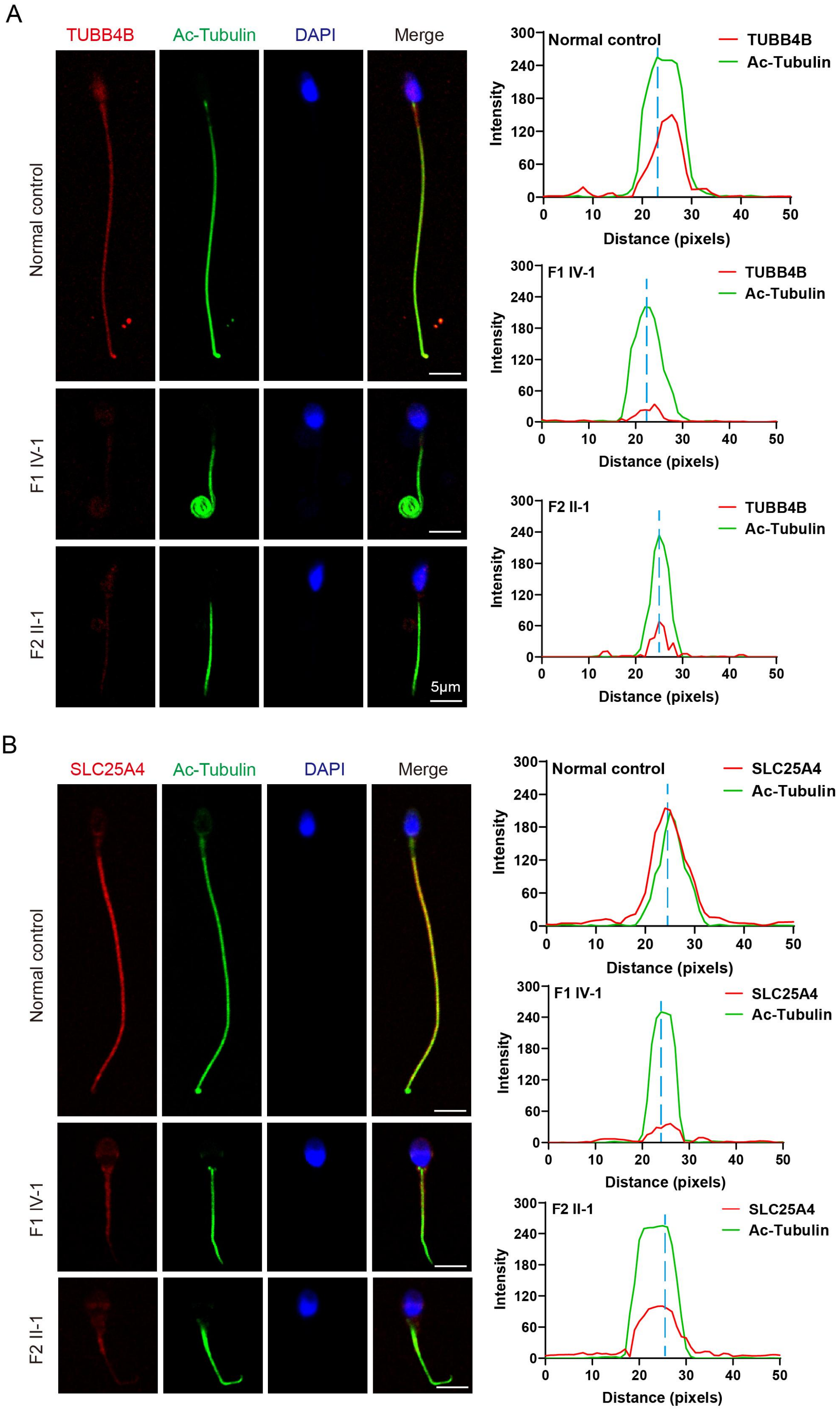

Figure S9. Reduced expression of TUBB4B and SLC25A4 in spermatozoa from individuals carrying *CFAP251* variants

(A) Immunofluorescence staining of TUBB4B (red), acetylated α-tubulin (green),

and DAPI (blue) in spermatozoa from control and affected individuals (F1 IV-1, F2 II-1). Line-scan analyses (right) show reduced intensity of TUBB4B proteins in affected individuals compared with controls. Scale bar, 5 μm. (B) Immunofluorescence staining of SLC25A4 (red), acetylated α-tubulin (green), and DAPI (blue) in spermatozoa. Line-scan analyses (right) show reduced intensity of SLC25A4 proteins in affected individuals compared with controls. Scale bar, 5 μm.

Table S1. Primers used for verification of human *CFAP251* variants.

Primer names	Primer sequences (5'-3')
M1F (F1 IV-1）	TGTGGGAAAGTTTAGCCAGTCC
M1R (F1 IV-1）	AGCACAGTGGCAATGATAGCTC
M2F (F2 II-1）	CAACTACTCAGGAGGCTGAGGCAGGAGA
M2R (F2 II-1）	TGTGGCCGGCTCACCCCTCTAATCCCAGC

Table S2. Primers used for verification of mouse *Cfap251* mutation.

Primer names	Primer sequences (5'-3')
m-cfap251-F	CTGCCACAGTTAGACGACG
m-cfap251-R	CCTCAGCGGCTAGGACAAG

Table S3. Antibodies used in this study.

Antibodies	Species	application	Concentration	Company source	Cat #
CFAP251	Rabbit	IF,WB,IP	IF(1:200),WB(1:1000),IP(1:250)	Sigma	HPA040005
Rabbit-IgG		IP	IP(1:1000)	Beyotime	A7016
α-Tubulin	Mouse	IF	IF(1:200)	Cell signaling	3873S
FLAG	Mouse	WB, IP	WB(1:200),IP(1:100)	Abmrt	M20008
MYC	Mouse	WB	WB(1:1000)	Abmrt	M20002
GFP	Mouse	WB	WB(1:1000)	Abmrt	M20004
TUBB4B	Rabbit	IF,WB	IF(1:100),WB(1:500)	Boster	BM4264
SLC25A4	Rabbit	IF,WB	IF(1:200),WB(1:2000)	OmnimAbs	OM644193
Ac-tubulin	Mouse	IF	IF(1:400)	Sigma	T6793
β-Actin	Mouse	WB	WB(1:2000)	Proteintech	66009-1-Ig
GAPDH	Mouse	WB	WB(1:2000)	Proteintech	60004-1-Ig
CABYR	Rabbit	WB	WB(1:2000)	Proteintech	12351-1-AP
SPAG6	Rabbit	IF,WB	IF(1:400),WB(1:2000)	Sigma	HPA038440
RSPH3	Rabbit	IF,WB	IF(1:400),WB(1:2000)	proteintech	17603-1-AP
DNAI2	Rabbit	IF,WB	IF(1:400),WB(1:2000)	proteintech	17533-1-AP
ODF2	Rabbit	IF,WB	IF(1:400),WB(1:2000)	Sigma	HPA001874
RSPH1	Rabbit	IF,WB	IF(1:400),WB(1:2000)	Sigma	HPA017382
TOMM20	Rabbit	IF,WB	IF(1:400),WB(1:2000)	proteintech	11802-1-AP
SEPT4	Rabbit	IF	IF(1:200)	Affinity	DF13393
Alexa Fluor 647	Rabbit	IF	IF(1:400)	Yeasen	34213ES60
Alexa Fluor 488	Mouse	IF	IF(1:400)	Invitrogen	A21206
Hoechst		IF	IF(1:200)	Beyotime	C1022
PNA		IF	IF(1:200)	Vectorlabs	FL-1071-5

Table S4. Primers Used for RT-qPCR Analysis.

Primer names	Primer sequences (5'-3')
M-CFAP251-F	ATGCAGCTCAACTATGCAGTAAA
M-CFAP251-F	TGAACACCAAGTATCGCTTCTG
M-GAPDH-F	GGTTGTCTCCTGCGACTTCA
M-GAPDH-R	TGGTCCAGGGTTTCTTACTCC

Table S5. *CFAP251* Variants Identified,Semen Routine Parameters and Sperm Morphology of the Man Carrying *CFAP251* Variants

**Patient**	**F1 IV-1**		**F2 II-1**	
cDNA alteration	c.1535+1G>C		c.1269+2T>C	
Variant allele	homozygous		homozygous	
Variant type	splicing		splicing	
Allele frequency in human population				
1KGP	N/A		N/A	
ExAc_all	N/A		N/A	
gnomAD	N/A		N/A	
**Semen Parameters**	Sample 1	Sample 2	Sample 1	**Reference Values**
Semen volume (mL)	3.5	3.5	3.3	>1.5
Concentration (10^6/mL)	17.4	15.5	11.8[a]	>15.0
Progressive motility (%)	0[a]	0[a]	1.7[a]	>32.0
DFI (%)	N/A	N/A	19.99[a]	≤15
**Sperm Morphology**				
Normal flagella (%)	6.8[a]		6.5[a]	>23.0
Coiled flagella (%)	52.7[a]		53.1[a]	<17
Short flagella(%)	25.9[a]		25.7[a]	<1.0
Absent flagella(%)	14.6[a]		14.6[a]	<5.0
Angulation(%)	0		0.1[a]	<13.0
Irregular caliber (%)	0		0	<2.0

NCBI reference sequence number of *CFAP251* is NM_144668.5

Abbreviations: DFI DNA fragmentation index, N/A Not applicable

[a] Abnormal values.

Table S6. ICSI outcomes of sperm from *Cfap251*$^{-/-}$ male mice.

	**WT**	***Cfap251***$^{-/-}$
**ICSI outcomes**		
No. of MII oocytes	73	52
Fertilization rate (%)	88(64/73)	40(21/52)
Cleavage rate (%)	98(63/64)	100(21/21)
Blastocyst formation rate (%)	53(34/64)	48(10/21)
